# Subtle Changes in Motif Positioning Cause Tissue-Specific Effects on Robustness of an Enhancer's Activity

**DOI:** 10.1371/journal.pgen.1004060

**Published:** 2014-01-02

**Authors:** Jelena Erceg, Timothy E. Saunders, Charles Girardot, Damien P. Devos, Lars Hufnagel, Eileen E. M. Furlong

**Affiliations:** 1 Genome Biology Unit, European Molecular Biology Laboratory (EMBL), Heidelberg, Germany; 2 Cell Biology and Biophysics Unit, European Molecular Biology Laboratory (EMBL), Heidelberg, Germany; University of California Berkeley, United States of America

## Abstract

Deciphering the specific contribution of individual motifs within *cis*-regulatory modules (CRMs) is crucial to understanding how gene expression is regulated and how this process is affected by sequence variation. But despite vast improvements in the ability to identify where transcription factors (TFs) bind throughout the genome, we are limited in our ability to relate information on motif occupancy to function from sequence alone. Here, we engineered 63 synthetic CRMs to systematically assess the relationship between variation in the content and spacing of motifs within CRMs to CRM activity during development using *Drosophila* transgenic embryos. In over half the cases, very simple elements containing only one or two types of TF binding motifs were capable of driving specific spatio-temporal patterns during development. Different motif organizations provide different degrees of robustness to enhancer activity, ranging from binary on-off responses to more subtle effects including embryo-to-embryo and within-embryo variation. By quantifying the effects of subtle changes in motif organization, we were able to model biophysical rules that explain CRM behavior and may contribute to the spatial positioning of CRM activity *in vivo*. For the same enhancer, the effects of small differences in motif positions varied in developmentally related tissues, suggesting that gene expression may be more susceptible to sequence variation in one tissue compared to another. This result has important implications for human eQTL studies in which many associated mutations are found in *cis-*regulatory regions, though the mechanism for how they affect tissue-specific gene expression is often not understood.

## Author Summary

Transcription is initiated through the binding of transcription factors (TFs) to specific motifs that are dispersed throughout the genome. Genomics methods have helped to discern which motifs for a TF are occupied and which are not, yet it is poorly understood why certain combinations of bound motifs, and not others, drive specific patterns of expression. Here, we take a bottom-up approach to address this question: We constructed simple, synthetic elements containing motifs for only one or two TFs in different orientations and integrated them into the *Drosophila* genome. By assessing when and where these elements drive expression, we could model specific rules governing tissue-specific enhancer activity. Despite the general importance of TF combinatorial interactions during development, elements with a single TF's motif were often sufficient to drive complex expression. By combining motifs for two factors, we observed non-additive expression in the heart. While the enhancer's activity could tolerate changes in motif spacing and orientation in many tissues, the robustness of heart expression was very sensitive to subtle sequence changes. These results highlight an important property of enhancers—as their readout is context-specific, so too are the effects of mutations within them, including small insertions that may alter a gene's expression in one tissue, but not in another.

## Introduction

Gene expression is initiated by the binding of transcription factors (TFs) to *cis*-regulatory modules (CRMs) such as enhancer elements, which give rise to specific patterns of temporal and spatial activity [1]. Recent years have seen a dramatic increase in the ability to identify the location of regulatory elements using genome-wide information on TF occupancy [2], [3], [4], [5], [6], [7], cofactor recruitment [8], [9] and chromatin modifications [10], [11], [12], [13], [14], [15]. These studies have identified thousands to hundreds-of-thousands of regulatory elements that could be potentially active at a given time and in a given cell-type during development. Given this extensive regulatory landscape, it has become an enormous challenge to decode how CRMs function in terms of their spatio-temporal activity. Detailed dissection of individual elements has revealed developmental enhancers whose function is dependent on the presence of individual TF motifs [16], [17], combinations of motifs [18], [19], [20], and the specific arrangement [21], [22], [23], [24], [25], [26] or not [2], [27], [28], [29] of those motifs. These examples have inspired much debate over the relative importance of each of these variables to enhancer function, but systematic rules to understand their contribution have not emerged.

While dissection of endogenous enhancers has proved to be an extremely powerful approach to understand enhancer function [19], [20], [29], [30], [31], [32], individual enhancers instantiate only one of many possible solutions that can give rise to a specific gene expression pattern [7], [28], [33], thereby limiting the range of functional rules that are generally explored. Synthetic elements offer the possibility to examine a wide range of motif compositions and motif positioning rules while ensuring, as much as possible, the neutrality of non-motif (*i.e.* spacer) sequences. Synthetic promoter-YFP libraries in yeast, for example, were used to quantify and model the effect of different promoter motif configurations on the levels of YFP expression [34], [35]. Similarly, synthetic constructs combined with massively parallel sequencing were used to dissect the relationship between DNA sequence and activity of constructs transiently transfected into cell lines [36] or injected into mouse tail veins [37]. These approaches offer the clear advantage of scale, as thousands of elements with different DNA sequence combinations can be examined simultaneously. However, they are limited by the simplicity of the read-out, which is a relative measure of the levels of the CRM's activity at a single time point or condition. For most developmental enhancers, the impact of sequence changes on the timing or tissue-specificity of gene expression is equally important. It is also not clear to what extent episomal DNA from transient transfection or tail-vein injections recapitulates the impact of chromatin context and nucleosome positioning on gene expression. In these respects, reporter assays in transgenic animals provide invaluable information. Although generally difficult to scale, multi-stage assays using stable transgenic embryos yield precise information about when and where an enhancer is active in an *in vivo* chromatinized context.

In this study, we systematically engineered 63 synthetic elements that differ with respect to the number and kinds of TF motifs they contain, as well as the relative spacing and orientation of these motifs. These elements were specifically designed to assess three properties of enhancer motif organisation relevant to metazoan development: (1) the ability of homotypic clusters of individual motifs to function as developmental enhancers, (2) the ability of combinations of different kinds of motifs (heterotypic motif clustering) to generate new emergent activity, and (3) the effect of changes in motif organisation, such as number, spacing and orientation, on the robustness of enhancer activity in both space and time. While many studies have focused on the effect of point mutations on enhancer activity, there is an enormous amount of structural variation within natural populations, including the sequence of *Drosophila* [38], [39] and humans [40]. To assess the influence of small insertions or deletions, we have systematically changed the spacing between motifs for four heterotypic pairs of TF motifs.

To examine these properties, we focused on ten motifs recognized by TFs that form part of a highly studied regulatory network that governs *Drosophila* mesoderm development [41], [42], including the downstream effector TFs of Wingless (known as Wnt in vertebrates) and Dpp (BMP in vertebrates) signaling pathways. We generated very simple elements consisting of six motifs in either a homotypic or heterotypic combination, which were stably integrated into the *Drosophila* genome. The resulting patterns of expression driven by these elements suggest a number of interesting features governing CRM function. First, despite the known importance of combinatorial activity to refine spatial patterns of expression, elements with multiple copies of an individual motif *can* drive complex patterns of expression. For example, although pMad is known to act cooperatively with lineage TFs in both *Drosophila* and vertebrates, it is sufficient to transduce Dpp activity when its motifs are in the appropriate configuration. Second, combining motifs for as few as two TFs can lead to novel emergent expression. Although the importance of cooperative DNA binding in the regulation of development has long been supported by other studies, the sufficiency of so few sites speaks to the extent to which even minor changes in *cis*-regulatory sequence can lead to the evolution of novel expression profiles. Third, the spacing and orientation of motifs is not only essential for enhancer activity in terms of binary on-off effects, but also has more quantitative effects on the robustness of gene expression, including inter- and intra- embryo variability. Fourth, these effects of motif organization, often referred to as motif grammar, are tissue-specific. The same ‘two-TF’ enhancer can function using very flexible motif spacing in one tissue, yet have rigid constraints in another, demonstrating an additional way in which the organization and function of CRMs acts to reduce the constraints of pleiotropy for regulatory mutations.

## Results

### Design of synthetic developmental enhancers

For each factor, synthetic CRMs were generated by combining six motifs, separated by a spacer sequence of defined length. Therefore, there were two aspects to the design of the synthetic elements; the choice of motif instance used for each TF and the sequence of the spacer. First, for the TF motifs, we selected high affinity motifs for each factor, as much as possible. TFs often recognize the same or highly similar sequences. This includes not only members of the same family of TFs, e.g. GATA factors (such as Pnr), but also TFs with apparently unrelated DNA binding domains, e.g. the bHLH factor Twist and the zinc finger TF Snail. While we cannot change this inherent property of TFs, we did try to increase the potential specificity of the motif (or word instance) used here for the particular TFs we are interested in by using motifs derived from *in vivo* occupancy data for nine of the ten factors [2], [7]. For clarity, we refer to each construct by the name of the TF from which ChIP data was used to learn the motif. However, the sequence of all motif instances used, as well as their similarity to other TF motifs is provided in Table S1.

**Figure 1:**
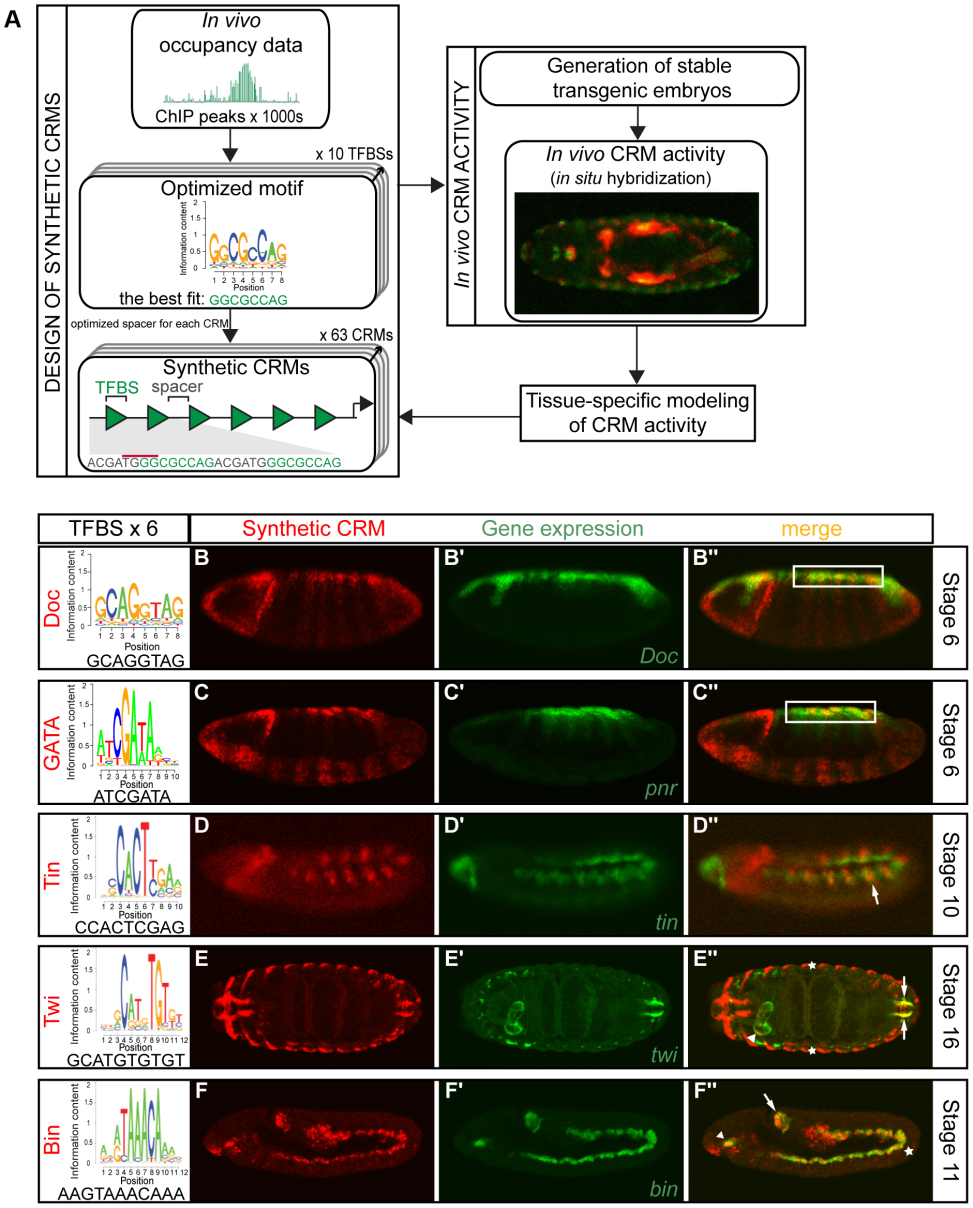
Simple synthetic elements containing homotypic TF motifs can generate complex spatio-temporal activity. (A) Schematic representation of flow from design of synthetic CRMs, assessment of *in vivo* activity to tissue-specific modeling. Optimized TFBSs (from ChIP experiments) were separated by spacers, with minimal affinity to known TF binding models, and placed in front of a minimal promoter and *lacZ* reporter and integrated into the *Drosophila* genome. (B–F) Homotypic synthetic CRMs containing six TFBSs from the represented sequence logo for each TF, separated by a 6 bp spacer. CRM activity was assessed by double fluorescent *in situ* hybridization of the *lacZ* reporter gene driven by synthetic CRM (red) and the corresponding endogenous gene (green). Synthetic CRMs composed from GATA (B–B″) and Doc (C–C″) motifs drive expression in the presumptive amnioserosa (white box). Tin synthetic CRM (D–D″) is expressed in the dorsal mesoderm (arrow), while Twi CRM (E–E″) is expressed in the foregut weakly (white arrowhead), hindgut (white arrows) visceral mesoderm and ectoderm (asterisks). Bin synthetic CRM (F–F″) is active in the foregut (arrowhead), midgut (asterisk) and hindgut (arrow) visceral mesoderm (VM). All embryos are shown laterally with anterior to the left, except for (E) which is a dorsal view.

Second, we tried to select a neutral spacer sequence that does not include known TFBS, based on our current knowledge. This is not trivial, as in addition to the spacer sequence itself, the border sequence bridging the spacer and the known motif (red bar, Figure 1A) can in itself create an additional binding site. To minimize this possibility, rather than using a common spacer, we computed an optimal spacer sequence for each combination of motifs to minimize the chance of inadvertently generating additional binding sites, based on current information (Supplemental Methods (Text S1)). During the course of this study, our results show that the spatio-temporal activity of the designed elements is primarily driven by the intended TF motifs and not from the spacer sequence, as indicated by two lines of evidence: (1) The concordance between the activities of homotypic and heterotypic elements for the same TFs, which were designed with different spacer sequences (described below) and 2) the very similar spatio-temporal activity of two heterotypic CRMs, which were specifically designed with identical TF motifs but with two different spacer sequences (pMad-Tin, see below).

Each synthetic CRM, which were on average 105 bp in length, was cloned into a common minimal *lacZ* reporter vector and stably integrated into the same location in the *Drosophila* genome using the phiC31 system [43], to allow for a direct comparison of enhancer activities. The ability of each CRM to drive spatio-temporal *lacZ* expression was assessed during all stages of embryogenesis by fluorescent *in situ* hybridization to examine the full regulatory potential of the DNA sequence.

### The regulatory potential of homotypic CRMs for different types of TFs

The combinatorial binding of TFs provides complexity to regulate refined spatial patterns of expression, and forms the basis for logical operations within Gene Regulatory Networks driving development [44]. However, in addition to combinatorial activity, homotypic clusters of an individual TF's motif are also present in regulatory regions in the vicinity of developmental genes in both *Drosophila* [45] and vertebrates [46]. Although prevalent *in vivo*, the role of these clusters in regulating gene expression, and the properties governing how they function, is currently not clear. Studies examining the ability of clusters of motifs to regulate gene expression in transgenic reporter assays have had varied success: this includes (although not exhaustive) homotypic clusters of motifs that were sufficient to drive activity [47], [48], [49], [50], [51], [52] and those that were not [48], [53], [54], [55], [56]. However, as the elements in each of these individual studies were designed and tested in different ways, it is impossible to deduce any functional inference across studies.

**Figure 2:**
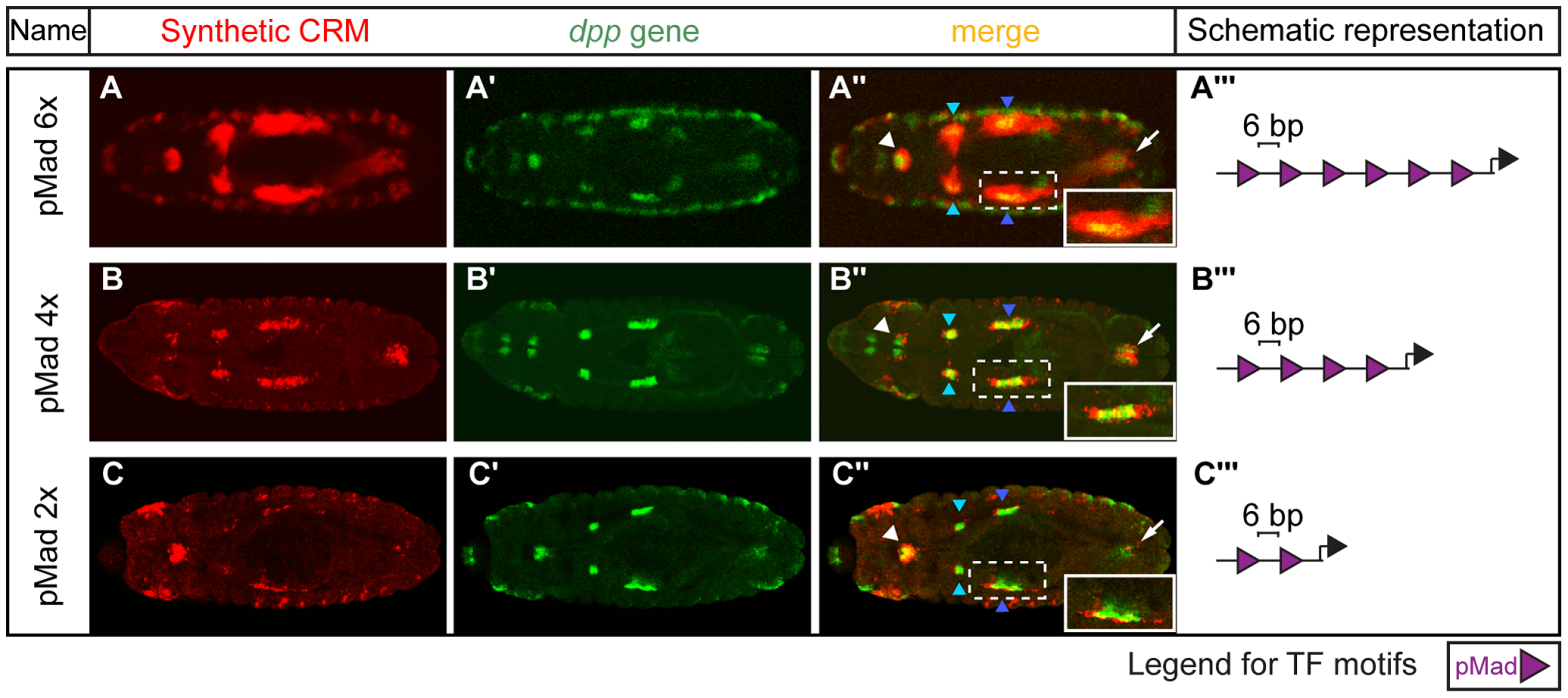
Altering the number of pMad motifs affects visceral mesoderm activity. Double *in situ* hybridizations against (A–C) the *lacZ* reporter gene driven by the pMad homotypic synthetic CRMs (red) and (A′–C′) the endogenous *dpp* gene (green). (A″–C″) Expression patterns in the foregut (white arrowhead); hindgut (arrow); parasegment 3 (PS3) (light blue arrowhead) and 7 (PS7) (dark blue arrowhead) of the midgut visceral mesoderm (VM). Dashed box is enlarged in the inset (white box). Embryos are dorsally oriented, with anterior to the left, stage 13/14. (A′″–C′″) Schematic representations of the CRM composition, where purple triangles depict the number and orientation of pMad sites with indicated spacing (bp) between adjacent sites.

We initiated this study by systematically examining the activity of elements composed of a cluster of six identical motifs. In total we tested homotypic CRMs with motifs for ten essential factors, encompassing multiple types of DNA binding domains: bHLH (Twist (Twi)), homeobox (Tinman (Tin), Bagpipe (Bap)), T-box (Dorsocross (Doc)), GATA zinc finger (Pannier (Pnr)), MADS box (Myocyte enhancer factor 2 (Mef2)), FoxF (Biniou (Bin)), HMG-domain (T-cell factor (TCF)), Ets-domain (Pointed (Pnt)) and the MH1 domain (pMad). Seven out of ten of these very simple elements were able to drive sequence-specific spatio-temporal expression (Figures 1B–F, 2, S1). In six cases, the expression profiles driven by clusters of a single motif were sufficient to partially (and in the case of two, almost completely) recapitulate the expression of the TF whose *in vivo* occupied motif was used to construct the CRM. For example, the synthetic CRMs containing Doc enriched and GATA motifs drove expression in the presumptive amnioserosa, cells where the endogenous *Doc* and *pnr* genes are expressed (white boxes, Figure 1B″,C″). Similarly the synthetic CRM containing Tin motifs drove expression in a subset of the dorsal mesoderm, colocalizing with the expression of the endogenous *tin* gene (Figure 1D″, arrow). Although multimerizing the preferred Twist E-box motif was not sufficient to drive mesoderm expression at early stages of development, this synthetic CRM could activate expression in the hindgut visceral mesoderm (VM) at later stages, overlapping the endogenous *twist* gene's expression (Figure 1E″, arrow).

The activity of two homotypic CRMs, containing motifs for either pMad (discussed below) or Bin, stood out as they were sufficient to recapitulate almost the entire domain of the TF's activity. For the Bin CRM, this included the foregut, midgut and hindgut VM (Figures 1F″, S2) through multiple stages of development. This result suggests that motifs for this FoxF factor are sufficient to regulate expression throughout the VM at multiple stages of development, consistent with Bin's essential requirement for VM development [57] and extensive enhancer occupancy [7], [58]. In contrast to the six CRMs that gave ‘expected’ activity, the activity of the homotypic dTCF CRM only partially overlaps *wingless* expression, (the ligand that activates the cascade leading to dTCF activation). The CRM's activity is restricted to segmental groups of cells that are in close proximity to, but not always adjacent to, the *wg* stripes (Figure S1A″), which may reflect more ‘long’-range signaling from Wg, or alternatively the activity of an additional TF that can occupy this dTCF motif.

Taken together, these results indicate that homotypic clusters of an individual motif are often sufficient to regulate specific patterns of spatio-temporal activity, analogous to the more commonly studied combinatorial elements. Multimerizing single TF motifs can provide remarkable specificity, as demonstrated by the non-random overlap of CRM activity with part or all of the TF's expression in six out of ten cases tested. Conversely, it is interesting that not all activator clusters are sufficient to drive expression, in contrast to what might be assumed from the activity of yeast GAL4 sites. For example, three synthetic CRMs (multimers of Pnt, Mef2 and Bap motifs) yielded no activity (Figure S1B–D). In the case of Mef2 and Twist, the lack of general mesoderm activity is surprising given that both TFs have very well characterised DNA binding specificities in both *Drosophila* and vertebrates. This diversity in CRM output may reflect inherent differences in the regulatory potential among different TFs or in their ability to act cooperatively in a homotypic manner to potentiate their activity.

### The extent of CRM activity scales with the number of TF motifs

The *dpp* gene, coding for the ligand of the Dpp signaling cascade, is expressed in the foregut, hindgut and midgut VM in parasegment 3 (PS3) and 7 (PS7) at stage 13/14 of embryogenesis [59]. *dpp* expression is restricted to these VM domains through the integration of activating inputs from Ubx and Bin [57], [59], and repression via Wg signaling [60]. The downstream effector TF of Dpp signaling is the phosphorylated form of Mad (pMad). Our synthetic CRM containing six pMad motifs could recapitulate almost the entire expression of *dpp* in the VM during these stages of embryogenesis (Figure 2A). Therefore, despite the complexity in the network of upstream factors regulating *dpp* expression, once *dpp* is expressed, sites for its downstream effector (pMad) alone are sufficient to activate enhancer activity in these sub-tissue domains. The spatial boundaries of the enhancer's activity are most likely refined through the action of Brinker (Brk). Brk is a transcriptional repressor whose expression is negatively regulated by Dpp signaling [61]. This results in cells with high levels of pMad and low Brk (Dpp responding cells, where our synthetic CRM is active) and those with high Brk and low pMad (neighbouring cells outside the Dpp signaling domain, where our CRM is inactive). As Brk and pMad can recognize the same motif [62], there is often direct competition between the two TFs to regulate enhancer activity, where the relative levels of both TFs serve to limit the spatial domain of Dpp target gene expression, as nicely demonstrated for the endogenous *Ubx* enhancer [62].

The sufficiency of pMad motifs alone to activate enhancer activity was unexpected given previous studies in both *Drosophila* [56] and vertebrates [63], [64], indicating that Mad proteins bind to enhancers cooperatively with other tissue-specific ‘lineage’ factors, and have little activity alone. We therefore further investigated the regulatory potential of pMad sites in isolation by directly comparing the activity of elements containing six, four or two motifs, in the same orientation and with the same spacer sequence (Figures 2, S3). The expression of *lacZ* in PS7 of the midgut VM (Figure 2) is particularly informative of pMad activity: the Dpp signaling cascade activates pMad in an autocrine [65] and paracrine [66] fashion, which likely results in the highest levels of pMad activation in VM cells in PS7 and lower levels in adjacent parasegments. The homotypic CRM containing six pMad motifs reflects this paracrine signaling, having a larger domain of expression covering neighboring cells compared to the *dpp* expressing cells (Figure 2A). In contrast, the synthetic CRM containing four motifs had more restricted activity to a narrow domain anterior and posterior to the *dpp* expressing cells (Figure 2B), while the CRM containing only two motifs was active only in the Dpp producing cells (autocrine signaling) (Figure 2C). Therefore, pMad sites alone, when present in close proximity, are sufficient to activate enhancer activity in the absence of specific lineage TFs. However, the extent of the activity is dependent on the number of available pMad motifs and on the balance between pMad and Brk concentration.

In addition to the number of cells in which the enhancer was active, we also observed a strong correlation between the number of sites and the strength of the CRM. In the amnioserosa, for example, the CRM with six pMad sites drove robust expression at stage 11 (Figure S3A″), while four sites resulted in reduced activity, and the CRM with two sites drove only very weak expression in the amnioserosa (Figure S3B,C). This trend was even more dramatic at stage 14, when the pMad concentration in the amnioserosa decreases [67]. While the CRM with six pMad sites drove strong activity at stage 14 (Figure S3D), the CRMs with either four or two sites were inactive in the amnioserosa at this stage. The number of pMad sites, thus, appears to be able to compensate for a decrease in the amount of accessible pMad protein at stage 14, presumably by providing a larger platform for cooperative binding.

### Changing motif organisation within a CRM affects its activity in a tissue-specific manner

Cooperative interactions between TFs can sometimes occur through direct protein-protein interactions (PPI), which may introduce constraints in the organization of the TFs' motifs within CRMs [1]. Taking advantage of the design flexibility of synthetic elements, we generated heterotypic CRMs composed of motifs for TFs known to exhibit protein-protein or genetic interactions: namely motifs for Tin with either Pnr, Doc, dTCF, or pMad [68], [69], [70], [71], to systematically explore two properties: 1) The influence of changes in the relative spacing and orientation of motifs on the robustness of CRM activity in different tissues, and 2) The ability of different combinations of TF motifs to generate emergent expression patterns in a particular tissue, not observed for CRMs containing only one kind of motif.

Each heterotypic CRM contained three pairs of TF motifs for either Doc-Tin, GATA-Tin, dTCF-Tin, or pMad-Tin. For each TF pair, on average nine constructs were tested in transgenic animals, in which the spacing and/or orientation of the Tin motif was systematically altered (Table S2). Heterotypic CRMs containing GATA-Tin or Doc-Tin combinations resulted in a pattern of expression that was largely the sum of the activity of each of the corresponding homotypic CRMs alone (data not shown). Changing the spacing and orientation of the sites (Table S2) had little or no effect on CRM activity, indicating a minimal role for motif positioning, or grammar, in these instances. The dTCF-Tin heterotypic CRMs did not have any activity, despite the fact that the homotypic CRMs containing six dTCF or Tin sites drove specific activity in segmental groups of cells in the ectoderm and mesoderm, respectively (Figures 1D, S1A). This lack of activity in the heterotypic context most likely reflects the reduction in the number of motifs used, from six in the homotypic constructs to three motifs for each factor in the heterotypic CRM. Correspondingly, homotypic construct containing four dTCF sites [53], [54] had no embryonic activity.

**Figure 3:**
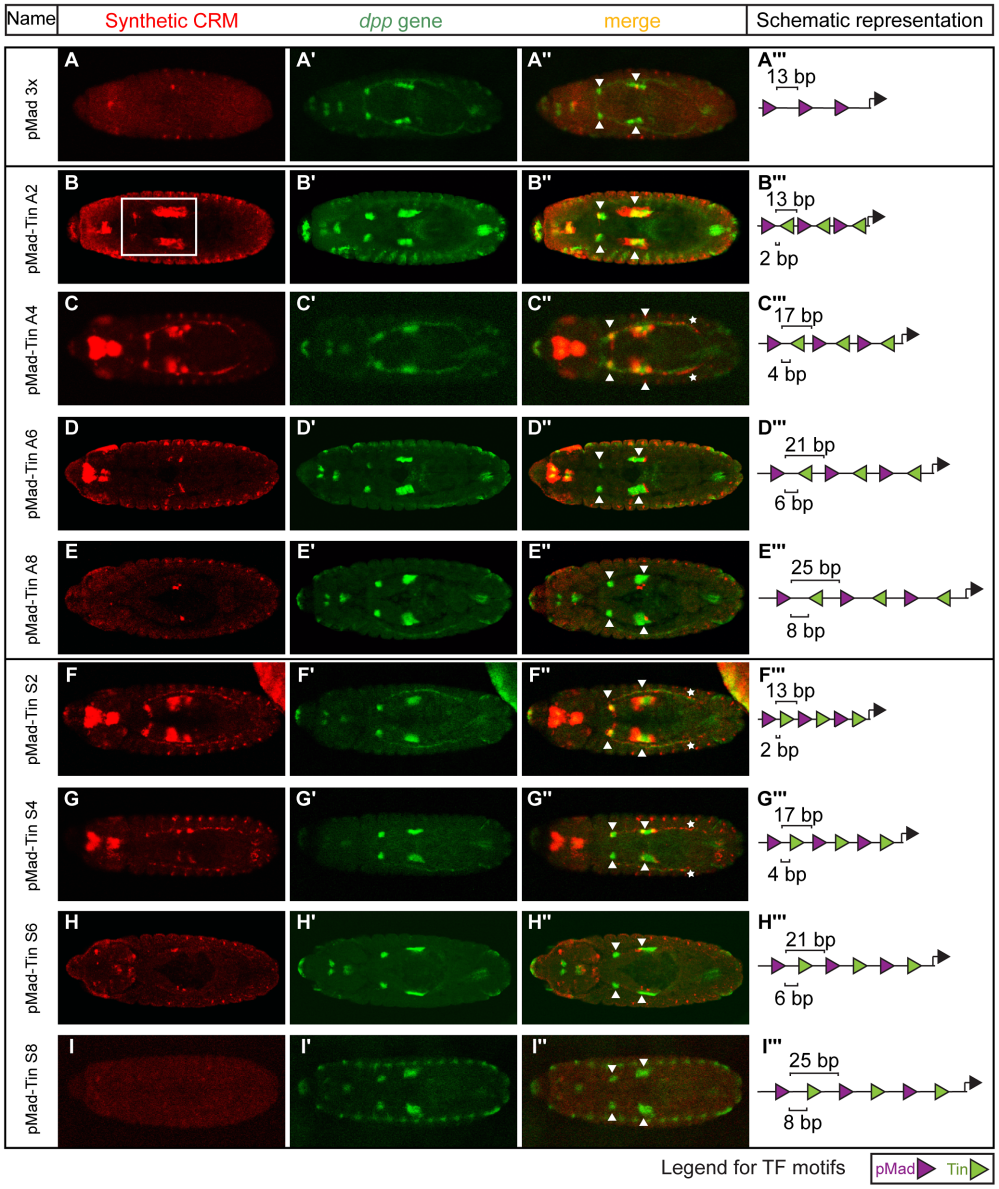
Changes in motif organisation have a graded effect on activity of CRMs in the visceral mesoderm. Double *in situ* hybridization against the (A–I) *lacZ* reporter gene driven by the synthetic CRMs (red) and (A′–I′) the endogenous *dpp* gene (green). (A″–I″) CRM activity in midgut visceral mesoderm is indicated with arrowheads or white box (B) and in heart with the asterisks (C″, F″, G″). Embryos are dorsally oriented, with anterior to the left, stage 13/14. (A′″–I′″) CRM composition, where triangles (pMad – purple, Tin – green) depict the number and orientation of sites. Spacing between adjacent pMad-Tin sites (below) and pMad-pMad sites (above) is indicated.

In contrast to the GATA-Tin and Doc-Tin heterotypic pairs, whose activity was robust to changes in motif organisation, the activity of the pMad-Tin heterotypic CRMs changed depending on the motif spacing. As discussed above, pMad motifs alone are sufficient to regulate expression in PS3 and PS7 of the midgut VM. In these cases, the strength of activity was dependent on the number of motifs present, with a dramatic reduction in expression observed going from four to two sites (Figure 2). A homotypic CRM containing three pMad sites separated by 13 bp also showed a dramatic reduction in VM activity (Figure 3A), a result that may stem from either or both the reduction in sites or the increase in spacing (from 6 bp to 13 bp). Interestingly, when a Tin motif is inserted within each 13 bp ‘spacer’ sequence, this pMad-Tin heterotypic design can restore activity in the midgut VM (Figure 3B, white square), giving a pattern of expression similar to the pMad homotypic CRM with 6 bp spacing. The ameliorative effects of the Tin sites were limited by distance. Increasing the spacing between adjacent pMad and Tin motifs from 2 to 8 bp, which increases the spacing between pMad motifs from 13 to 25 bp, caused a progressive reduction in *lacZ* expression in the midgut VM (Figure 3). This VM activity is primarily driven through the pMad and Tin motifs and not the spacer sequence, as changing the spacer sequence had a minimal effect on VM enhancer activity, while a similar distance effect was observed by altering the length of spacer between the two sites (compare Figures 3 and S5). These results suggest that the occupancy of Tin sites acts either to bridge the distance between pMad sites (up to ∼20 bp) facilitating cooperative pMad regulation or alternatively to facilitate pMad-Tin combinatorial regulation of VM expression.

To distinguish between these two possibilities, we examined the effect of altering the relative orientation of the sites, reasoning that if Tin occupancy acts indirectly to help pMad recruitment altering the orientation of the Tin site should have little effect on activity. However, we observed that changes in motif orientation did influence CRM activity: one orientation of the Tin motif (arbitrarily referred to as antisense (A)) typically drove stronger VM expression compared to CRMs with the motif in the opposite orientation (referred to as sense (S)) (Figure 3, compare C to G). This result indicates that the Tin motifs are not just neutral sequences bridging the spacing between homotypic pMad motifs, but rather suggest a specific mechanism of cooperative Tin-pMad DNA binding, and therefore transcriptional regulation.

### Non-additive activity by combining two different motifs: Emergence of heart activity

**Figure 4:**
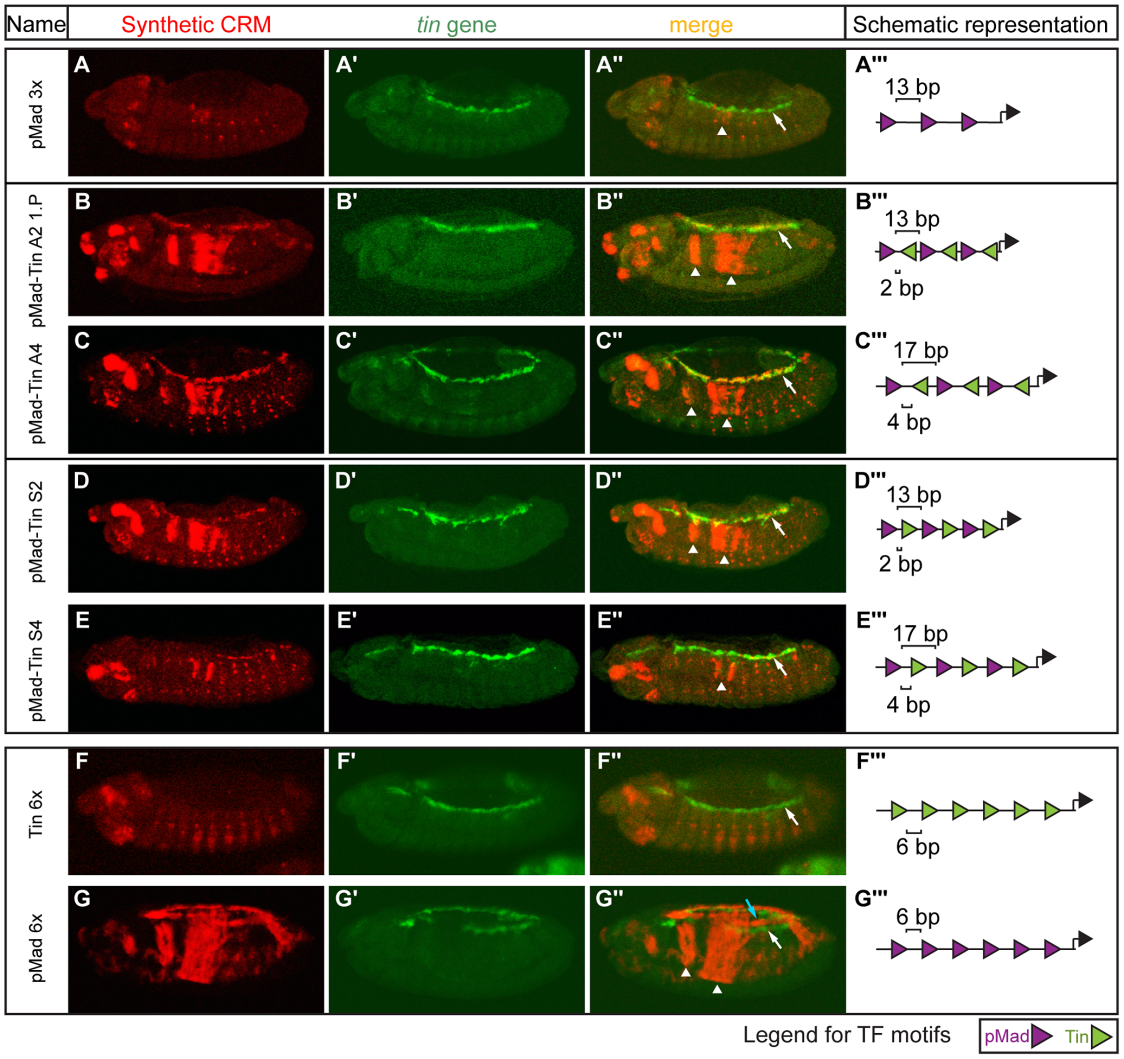
Emergence of heart expression by combining two TFs' motifs in limited configurations. Double *in situ* hybridization against the (A–G) *lacZ* reporter gene driven by the synthetic CRMs (red) and (A′–G′) the endogenous *tin* gene (green). (A–A″) pMad 3× CRM with 13 bp spacer has no heart activity (white arrow) and a severe reduction in visceral mesoderm activity (arrowhead). (B″–E″) pMad-Tin CRMs containing 2 and 4 bp spacers were active in the heart (white arrows) as indicated by colocalization with *tin* gene. (F″,G″) CRMs composed of only Tin or only pMad sites were unable to drive expression in heart. Expression in the midgut visceral mesoderm is indicated with arrowheads (A″–E″, G″) and in amnioserosa with light blue arrow (G″). Embryos are laterally oriented, with anterior to the left, stage 13/14. (A′″–G′″) CRM composition, where triangles (pMad – purple, Tin – green) depict the number and orientation of sites. Spacing between adjacent pMad-Tin sites (below) and pMad-pMad sites (above) is indicated.

Combining pMad and Tin sites resulted in CRM activity in the dorsal mesoderm, VM and amnioserosa –representing essentially the summation of the expression profiles of the respective homotypic CRMs (Figures 1D, 2A, S4). However, in contrast to GATA-Tin and Doc-Tin CRMs, whose joint expression profiles were largely additive, the heterotypic pMad-Tin CRMs were also sufficient to drive expression in a new domain, the developing heart (Figure 4). Cardioblast specification requires the action of a large number of TFs and signaling cascades (for review, see [72]). Tin, Pnr (GATA factor), and Doc together with Dpp and Wg signaling are essential for heart development [73], [74], [75], [76], [77], [78], a tissue in which these factors regulate each others' expression [70], [75], [76], [77], [78], [79], [80], and act as a collective unit to regulate enhancer activity [2], [27]. Given this complex regulation, we did not anticipate that a simple element containing only pMad and Tin sites would be able to drive expression in the heart. Importantly, the emergence of this pattern was highly dependent on the CRM architecture: Among our constructs, heart activity was only observed in CRMs containing three pMad and three Tin motifs placed in close proximity (within 2–4 bp) to one another (Figure 4B–E). In contrast, no heart activity was observed in CRMs where the spacing between motifs was increased to 6 bp or 8 bp.

Even in the ‘optimal’ motif configuration, we observed embryo-to-embryo variability, indicating that expression in the heart is significantly less robust (*i.e.* more influenced by stochastic events) than is expression driven by pMad-Tin constructs in the VM and other tissues. To better assess the robustness of these constructs activity, we made use of *P*-element transgenesis to integrate our CRMs into random locations in the genome. We reasoned that non-robust CRM activity would be more highly influenced by variation in chromatin context at different genomic positions compared to more robust expression profiles. Across these random-insertion sites, the pMad-Tin CRMs drove highly consistent expression in the VM, amnioserosa, and dorsal mesoderm, while heart activity in those same embryos varied dramatically as a function of genomic location (Table S3). In fact, the pMad-Tin A2 CRM was only active in the heart in the context of a transgenic fly line obtained using a *P*-element (Figure 4B), and not with the phiC31-mediated integration (Figure 3B). These results indicate that the heart activity of pMad-Tin CRMs is teetering on the edge of activation, being highly sensitive to both the motif context within the enhancer and the enhancer context within its chromatin environment. Thus, while the combined activity of these two TFs can give rise to emergent activity in the developing heart, this is not a robust mechanism to generate heart expression.

### Sensitivity to motif organisation varies in a tissue-specific manner

**Figure 5:**
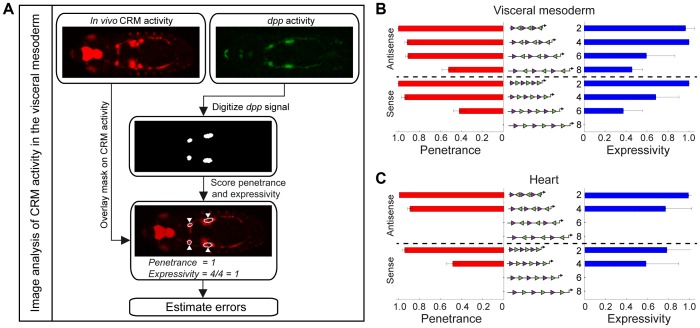
Quantifying the penetrance and expressivity of CRM activity. (A) Automated image analysis workflow. The gene expression pattern was digitized to create a mask for the tissue of interest. Comparing the mask with CRM activity enabled rapid and reliable scoring of both penetrance and expressivity. Errors in penetrance and expressivity were estimated as described in Supplemental Methods (Text S1). Penetrance (red bars) and expressivity (blue bars) of pMad-Tin heterotypic CRMs in the VM (B) and the heart (C). Note that *P*-element pMad-Tin A2 line was used to quantify heart activity.

Examining CRM activity in the heart revealed that some CRMs exhibit varied activity among embryos, and even within an embryo, as demonstrated by the pMad-Tin S4 construct, which drove expression throughout the entire heart in some embryos (Figure 3G), but only in a posterior portion of the heart in others (Figure 4E). To assess this variability in a systematic manner, and to provide quantitative data to which we could fit a model explaining CRM activity (see below), we applied two measures of the robustness of CRM activity: (1) *Penetrance*, defined as the fraction of embryos within a population that show CRM activity in the relevant tissue (in this case the VM or heart), and determined by any spatial overlap of *lacZ* expression with *dpp* (VM) or *tin* (heart) at a defined developmental stage (Figures 5A, S6A). (2) CRM *expressivity*, defined as the fraction of tissue-specific regions within an embryo (*i.e.* the proportion of the VM or heart) that display CRM expression (Figures 5A, S6A). In the midgut VM, for example, there are four domains of CRM activity (Figure 5A). If the CRM is active in all four domains, it has an expressivity of 1, while activity in two out of four domains has an expressivity of 0.5. To minimize systematic error, we implemented automated image analysis (see Materials and Methods) of embryos at a consistent stage of development (Figures 5A, S6A). In using penetrance and expressivity as quantitative metrics of CRM activity, we avoided issues arising from directly comparing non-linearly amplified signals from standard *in situ* hybridization of *lacZ* levels between CRMs. Penetrance provides a reliable measure of enhancer embryo-to-embryo variability, while expressivity provides a readout of intra-embryo enhancer variability – both of which we exploit to assess the effect of motif organization on the robustness of activity in two tissues.

The CRM penetrance of around one hundred embryos was measured for each of the 8 pMad-Tin synthetic CRMs (Table S4). With this quantification, differences in activity became more striking in several regards. First, within a tissue, subtle differences in activity between CRMs with different motif configurations become clear. For example, in the antisense orientation, changing the motif spacing from 2 bp to either 4 bp or 6 bp spacing resulted in only a slight decrease in penetrance, from 1 to 0.91 in the VM. However, the expressivity of these CRMs was quite distinct. The CRM with a 4 bp spacing had an expressivity of 1, while this number was nearly halved (0.59) for the CRM with a 6 bp spacing (Figure 5B; Table S5). The extreme effect of a small (2 bp) change in motif spacing suggests that direct, and potentially cooperative, interactions among bound factors have been disrupted, leaving the enhancer's activity more prone to variation.

A second striking observation is the extent to which changes in CRM architecture impacts activity in a tissue-specific manner. This is made clear by relative differences in CRM penetrance between the VM and heart. In the antisense orientation, for example, CRM penetrance remains almost unchanged in the VM when the motif spacing between the pMad and Tin sites is changed from 2 to 6 bp (Figure 5B). In contrast, the penetrance in the heart drops from 0.89 at a 4 bp spacing to zero when the motifs are spaced by 6 bp. A similar dramatic effect was observed by flipping the orientation of the Tin motif from antisense to sense, at a 4 bp spacing, which caused the penetrance of heart activity to drop from 0.89 to 0.48 (Figure 5C). These switch-like transitions in penetrance indicate that heart activity can only occur with a very restricted motif organization, which relies on close proximity of all TF motifs.

Taken together these results highlight an important property of *cis-*regulatory activity in multicellular organisms: An enhancer element (e.g. composed of only two types of motifs as described here) can require a very restricted motif configuration to regulate expression in one tissue (heart), but yet be much more flexible in its motif organisation to drive robust activity in another (VM).

### Modeling TF occupancy reveals tissue-specific rules of TF cooperativity

The extent to which cooperative interactions, including higher-order interactions across multiple proteins, contribute to enhancer activity is difficult to assess by simply visualizing expression patterns. Here, computational models can be extremely helpful to explore the effect of potential interactions on CRM function [33], [81]. To better understand the contribution of higher-order interactions among TFs on our synthetic CRMs, we used fractional site occupancy modeling (Figures S7; Methods (Text S1)), which describe DNA-protein and protein-protein interactions as thermodynamic processes, an approach that has been successfully used to understand other regulatory elements [82], [83], [84], [85], [86], including *Drosophila* enhancers [26], [87].

As fractional site occupancy models analyze the probability of every possible configuration of binding events, the complexity of the models increases exponentially with the number of TFBSs. To avoid these complications, we aimed to identify the simplest model that recapitulates the observed CRM activity. In line with our observations (as seen in Figures 3 and 5), and with research in another tissue [71], the model assumes the presence of direct cooperative interactions between neighbouring pMad and Tin proteins. Protein-protein interactions were modeled as the extent of overlap between spheres of “interaction space” around each bound protein, with sense and antisense orientations having different effective spherical radii (Figure S7, and includes parameters for the strength of possible cooperativity between bound TFs (Supplemental Methods (Text S1)). The model specifically explores whether additive interactions between Tin-pMad pairs are sufficient to recapitulate the observed experimental data or if potential ‘higher order’ cooperativity among pMad-Tin pairs with nearby pMad or Tin bound proteins are required to drive robust CRM activity.

**Figure 6:**
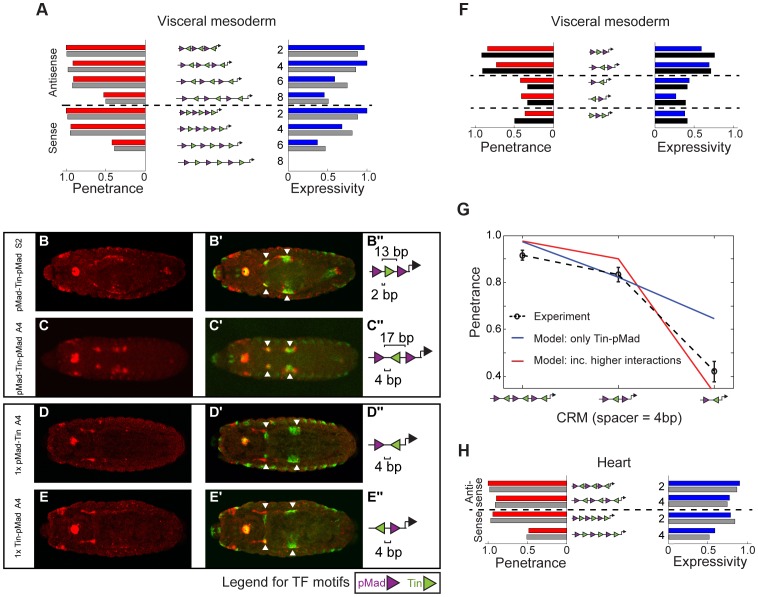
A fractional occupancy model learned different cooperative rules of TF binding that explain CRM penetrance and expressivity. (A) Red and blue bars represent the experimentally measured penetrance and expressivity, respectively, of the six-motif pMad-Tin heterotypic CRMs in the VM. Gray bars represent the model's fitted results when pMad-Tin-pMad cooperative interactions were included. (B–E) CRM activity for short pMad-Tin heterotypic CRMs. Double *in situ* hybridization against the *lacZ* reporter gene driven by the synthetic CRMs (B–E, red) and the endogenous *dpp* gene (B′–E′) green), where arrowheads indicate the midgut visceral mesoderm (VM). Embryos are dorsally oriented, with anterior to the left, stage 13/14. (B″–E″) Schematic representations of the CRM composition, where purple and green triangles depict the number and orientation of pMad or Tin sites, respectively. Spacing between adjacent pMad-Tin sites (below) and pMad-pMad sites (above) is indicated. (F) Penetrance (red bars) and expressivity (blue bars) of the short pMad-Tin heterotypic CRMs in the VM with the model's prediction (black bars) when pMad-Tin-pMad cooperative interactions were included. (G) Model predictions for pMad-Tin enhancers of different motif number with antisense orientation of the Tin site and 4 bp separation between sites. Red (blue) curve corresponds to the model prediction with (or without) pMad-Tin-pMad interactions. (H) As (A) but for the heart, where only non-zero results are shown. Note that we fit the heart data including the penetrance and expressivity results from the *P*-element pMad-Tin A2 line. Gray bars represent the model fit when considering Tin-pMad-Tin as the minimal cooperative TF configuration.

For VM activity driven by the pMad-Tin heterotypic CRMs, a model that includes an additional degree of cooperativity beyond pMad-Tin pairs fit the data better compared to a model in which pMad-Tin pairs act in an independent additive manner (Figures 6A, S8A). A key difference between the models lies in their predictions of the robustness of shorter CRMs: if higher order interactions are central for robust CRM activity then shorter CRMs will have a sharper decrease in robustness compared to pMad-Tin interactions alone. To experimentally test this, we halved the size of two heterotypic CRMs, generating CRMs with pMad-Tin-pMad motifs in S2 and A4 configuration. Such small CRMs could drive expression in the midgut VM (Figure 6B,C), albeit at a reduced level. In contrast, CRMs with only one pMad and Tin site, representing the smallest possible cooperative binding configuration (a pMad-Tin pair), drastically reduced all VM activity (Figure 6D,E). Incorporating higher level cooperativity into the model, without any further fitting, significantly improved the quality of the prediction of the shortened CRMs (Figures 6F,G, S8B). This suggests that pMad-Tin-pMad is the *minimal* configuration essential for *robust* VM expression. Importantly, this model was robust to a ‘leave-two-out’ cross validation iterated over all possible orderings of the 12 CRMs, arguing against over-fitting (Figure S8C). Finally, to test the model's prediction that additive interactions between pMad-Tin pairs with other bound pMad or Tin proteins are insufficient to drive robust VM expression, we tested the activity of a CRM with the motif configuration Tin-pMad-Tin. This CRM had very weak VM activity (Figure S8D), consistent with a requirement for higher order interactions between multiple pMad-Tin pairs for robust CRM activity (Figure 6G). In summary, increasing the complexity of TF cooperativity resulted in significantly improved consistency with experiment compared to considering only independent pMad-Tin cooperative pairs (Figure 6G).

Next, we addressed how heterotypic pMad-Tin CRMs lead to activity in the heart. Simple models with cooperativity between bound Tin and pMad can recapitulate the observed penetrance and expressivity in the heart for CRMs (Figures 6H, S8E,F). However, only the model including higher-order cooperative interactions between three neighboring units of Tin-pMad-Tin was consistent with both the shorter constructs and the six TF motif CRMs (Figures 6H, S8E,F). We note that the true minimal motif arrangement to generate robust heart activity is likely to be much more complex. In line with this, the Tin-pMad-Tin CRM has no heart activity. Interestingly, the effective range of cooperative TF interactions learned by the model for the heart was considerably lower (<5 bp) than for the VM (<9 bp) (Table S6).

In summary, while our experimental data is suggestive of cooperative TF interactions being likely necessary for CRM activity, the modeling has formalized this and systematically identified the range of interactions and the likely *minimum* level of higher-order TF cooperativity required for activity in both the VM and heart. Taken together, the modeling provides regulatory rules that explain how the same two TF motifs can give rise to activity in two different tissues (VM and heart) depending on the motif organization within the CRM.

## Discussion

In this study, we stably integrated simple synthetic CRMs into transgenic *Drosophila* embryos, and then combined a quantitative analysis of enhancer activity with fractional site occupancy modeling to determine the contribution of motif organization to activity in two tissues. The results of these analyses highlight a number of organizational features that contribute to an enhancer's activity during development.

### Tissue-specific sensitivity to motif organisation

**Figure 7:**
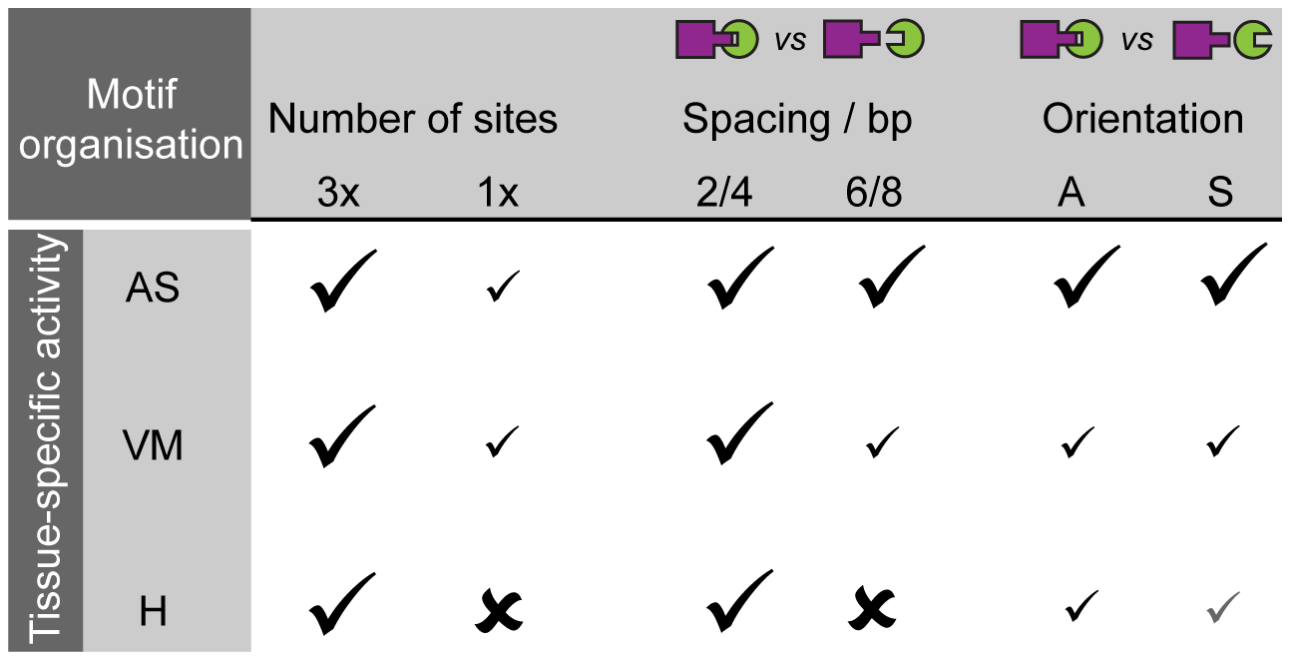
Motif organisation affects the ability of two TF motifs to elicit activity in a tissue-specific manner. Schematic table summarizing our experimental data of CRM activity for pMad-Tin CRMs, which display tissue-specific sensitivity to motif organisation (spacing, orientation) in different tissues: amnioserosa (AS), visceral mesoderm (VM) and heart (H). The effect is not always an on-off switch, but can produce a range of responses (depicted with differential strength of check marks), which affects robustness of CRM activity.

While quantifying the activity of a simple ‘two-TF motif’ CRM (pMad-Tin), our results show that enhancer activity can exhibit very different sensitivity to motif organization in one tissue compared to another (Figure 7). Several mechanisms could account for this interesting effect, including different concentrations of the TF (*i.e.* pMad or Tin) in the different tissues, the availability of tissue-specific co-factors, or tissue-specific priming of the enhancer, which may increase the ease by which the enhancer is activated.

An elegant dissection of the endogenous *spa* enhancer demonstrated that completely rearranging the relative order and spacing of TF binding sites could switch its cell type-specific activity from cone cells to photoreceptors in the eye [20]. In comparison, the changes in motif organisation introduced in our study were much more subtle such that the relative order of motifs was completely preserved. Yet only changing the spacing or orientation of motifs altered the robustness of enhancer activity in a tissue-specific manner. This result indicates that small insertions or deletions in CRMs, that do not affect the TF motifs themselves, could still have significant effects on gene expression in one tissue while having no effect in another. A study examining the activity of neuroectoderm enhancers between *Drosophila* species supports this model, where reduced spacing between Dorsal and Twist sites results in broader neuroectodermal stripes of CRM activity, while increased motif spacing resulted in progressively narrower stripes [88]. Studies of both endogenous enhancers and the synthetic CRMs described here provide compelling evidence that the exact positioning of motifs within CRMs is crucial for the robustness of their activity in one tissue, while it may be largely dispensable in another. Different cell types can therefore interpret the same motif content of a given enhancer in different manners.

### Non-additivity of CRM motifs: Variabile heart expression

The *Drosophila* heart is composed of two cell types, cardioblasts and pericardial cells, each of which requires the integration of many regulatory proteins for proper specification and diversification [72]. A characterized pericardial enhancer, *eve* MHE, for example, contains pMad and Tin binding sites in addition to sites for dTCF, Twi, Ets proteins, and Zfh1 [17], [89]. Given this complexity, it was surprising that a simple element built from pMad and Tin sites alone was sufficient to drive expression in the heart, albeit at a later developmental stage. Our analyses indicate that this activity is due to cooperativity binding between Tin and pMad, facilitated by a very specific motif arrangement. Using crystal structure data from close homologues of pMad [90] and Tin [91], we modelled the two TFs interaction on DNA, using a similar range of motif spacing (Figure S9). This 3D structural model indicates that it is possible for the DNA binding domains of these two proteins to both bind to DNA at a 2 bp spacing and to physically interact at a 2 bp and 4 bp spacing, but not at 6 bp spacing. Although done by homologue mapping, this structural data is consistent with our functional analyses of CRM activity, and further supports direct DNA binding cooperativity between these two TFs.

It is interesting to note, that although pMad and Tin sites are sufficient to drive expression in the heart from stage 13 to 14 (when placed in a limited motif arrangement), nature appears to use other enhancer configurations to regulate this critical function. There are two important aspects to this finding. First, heart activity arising from CRMs containing pMad and Tin sites alone is not robust. The enhancers are on ‘the edge’ of activation, where subtle changes in motif positioning or enhancer location switch activity between embryos and within embryos. Second, endogenous enhancers that are bound only by pMad and Tin – with no known input from other factors – direct expression in the dorsal mesoderm and not in the heart, at stage 10 [2], [92]. In our synthetic situation, pMad and Tin sites also drive robust expression in the dorsal mesoderm, in addition to variable weak expression in the heart. Therefore, although pMad and Tin sites alone are sufficient to drive heart activity in limited motif contexts, this mechanism is most likely not robust enough to be generally used to drive heart expression *in vivo*. This is consistent with recent studies showing that heart enhancer activity is elicited by the collective action of many TFs, which can occupy enhancers with considerable flexibility in terms of their motif content and configuration [2], [27]. Our pMad-Tin synthetic elements uncovered a very simple, although not very robust, alternative mechanism to regulate heart activity, and represent a nice example of how combinatorial regulation can lead to emergent expression profiles more than the simple sum of its parts.

### Robustness of CRM activity

The expression of key developmental genes is generally buffered against variation in genetic backgrounds and environmental conditions. This may occur at many levels including RNA polymerase II pausing [93], [94] and the presence of partially redundant enhancers [95], [96], [97], [98]. However, robust expression may also be buffered by the motif content within an enhancer to ensure a stable regulatory function. CRMs, for example, often include additional binding sites to those that are minimal and necessary [99]. In the context of the pMad-Tin synthetic CRMs, the motif organization can also act to ensure robust activity. Our results demonstrate that even in situations where the composition of motifs and their relative arrangement are maintained, subtle changes in the spacing between the motifs could have dramatic effects on enhancer output. Interestingly, this effect seems to be very tissue-specific, with some tissues maintaining robust activity whilst others lost all enhancer activity.

Taken together, the data presented in this study demonstrate that subtle alterations in motif organization can affect the ability of different tissues to ‘read’ an enhancer, which in turn may allow each tissue to fine-tune enhancer activity based on fluctuations in its molecular components.

## Materials and Methods

### Design of the sequence used in the synthetic CRMs

Binding affinity models (PWMs) for Twi, Tin, Mef2, Bap, Bin, dTCF, pMad, and Doc2 and GATA were derived from ChIP-chip data analyses [2], [7]. The model for Pnt was generated using published footprints (Supplemental Methods (Text S1)). PWMs were first trimmed on each side to remove positions with an information content (IC) of less than 0.4 (trimming stopped at the first IC position > = 0.4). The sequence that best fits the PWM model was then determined for each trimmed PWM and is referred to hereafter as “TFBS”. All TFBS sequences used to design the synthetic CRMs are available in Table S1. For each CRM, a ‘neutral’ spacer sequence (a linker sequence placed between motifs) was heuristically determined by minimizing the sequence affinity for known TF PWM models (Supplemental Methods (Text S1)).

### Cloning and transgenesis of the synthetic CRMs

Synthetic CRMs were generated from long oligonucleotides synthesized by Eurofins MWG Operon with compatible cohesive ends upon annealing for cloning. The forward and reverse strand oligonucleotides were phosphorylated, annealed and subsequently ligated into the pDUO2n [7], to generate stable, transgenic *Drosophila* lines using the phiC31 site-specific integrase [43]. The pH-Pelican [100] vector was use to test the robustness of enhancer activity at different genomic locations by random *P*-element transgenesis. The sequence of each CRM was verified to ensure that there were no synthesis errors and is provided in Table S2.

### In situ hybridization and microscopy

CRM activity was assessed in embryos from transgenic flies using fluorescent *in situ* hybridization as described previously [101]. The following ESTs or full length cDNAs from the *Drosophila* Gene Collection (DGC) were used to generate probes: RE13967 (*bap*), RE40937 (*doc2*), RE20611 (*dpp*), GM04312 (*dTCF*), SD02611 (*pnr*) and AT15089 (*twi*). cDNAs used for *bin* and *tin*, *lacZ*, *Mef2* and *pnt* were generous gifts from M. Frasch, U. Elling and M. Taylor respectively. Double or triple *in situ* hybridizations were performed using anti-fluorescein-POD, anti-DIG-POD and anti-biotin-POD antibodies (Roche, 1∶2000 dilution) and were developed sequentially with Cy3, fluorescein, and Cy5 tyramide signal amplification reagents (Perkin Elmer TSA kit). The *lacZ* expression patterns were imaged using Zeiss LSM 510 FCS or LSM 510 META confocal microscopes with A-Plan 10×/0.25 PH1 objective.

### Automated image analysis for the quantification of tissue expressivity

Background subtraction of both the CRM and tissue-specific channels was performed using a morphological opening with disk size greater than the largest relevant VM region (typical disk size of 25 pixel radius). The tissue-specific subset of images (e.g. *dpp* or *tin in situs*) were segmented using the Ilastik software package (www.ilastik.org). The segmented images were analysed using Matlab. The segmented regions in each image were smoothened by performing dilation (disk size of 5 pixel radius) followed by equivalent erosion. An area threshold (>200 pixels) was used to remove small, segmented regions. Finally, the perimeter of the segmented regions was calculated (using Matlab function *bwperim*) and overlaid onto the CRM expression data.

### Calculation of penetrance and expressivity

The penetrance was calculated for approximately 100 embryos for each of the twelve lines (Table S4). Bootstrapping was used to estimate the error in the penetrance measurements. The expressivity was calculated from around 16 carefully staged and positioned embryos (based on morphology and markers for VM (*dpp*) and heart (*tin*) tissues) for each line (Table S5). Embryos aligned dorsally were imaged and four regions of midgut VM were assigned, as shown in Figure 5A. The observed heart expression (also viewed dorsally) occurs in two rows of cells along either side of the embryo. We separated each row into an anterior and posterior segment, resulting in four heart regions (Figure S6A). The posterior segments, to the right of the PS7 VM region, correspond roughly to the heart proper, while the defined anterior heart segments correspond roughly to the region often referred to as the ‘aorta’. The penetrance in both the VM and heart was therefore measured as signal in one to four different regions of the tissue.

### Modeling of CRM activity

A fractional occupancy model was used to analyze the experimental data [102]. Our methodology was similar to other thermodynamic models used to understand CRM activity in *Drosophila* (e.g. [26], [87]). The model had at most four parameters: two parameters described the relevant protein-protein interactions; and two parameters were used to distinguish sense and antisense binding effects. Mathematical details are provided in the Supplemental Methods (Text S1).

### Ethics statement

This work is carried out in *Drosophila*, and was conducted in compliance with EMBL's guidelines.

## Supporting Information

Figure S1 Spatio-temporal activity of homotypic synthetic CRMs. All homotypic synthetic CRMs contain optimized TFBS from *in vivo* occupancy data, represented by the sequence logo for each TF, repeated six times and separated with a spacer of 6 bp. Double fluorescent *in situ* hybridization of the *lacZ* reporter gene driven by the synthetic CRM (A–D, red) and the corresponding TF's endogenous gene (B′–D′, green), or *wg* in the case of dTCF (A′). The dTCF CRM drives expression in segmental groups of cells adjacent to *wg* expression (A, A″). Pnt (B, B″), Mef2 (C, C″) and Bap (D, D″) synthetic CRMs did not drive any specific expression. All embryos are laterally oriented with anterior to the left.
(PDF)

Figure S2 A synthetic CRM with six Bin motifs is sufficient to drive expression throughout the visceral mesoderm. The Bin motif (represented by the sequence logo) was multimerized six times, separated by a 6 bp, to generate the homotypic synthetic Bin CRM. Double *in situ* hybridization against the *lacZ* reporter gene driven by the synthetic CRM (red) and the endogenous *bin* gene (green), showing expression in the forgut (arrowhead), midgut (asterisk) and hindgut (arrow) visceral mesoderm as indicated by the colocalization with *bin* (yellow) during embryogenesis. Background plasmid activity is depicted with a blue arrow. All embryos are lateral views with anterior to the left.
(PDF)

Figure S3 pMad homotypic CRM activity in the amnioserosa is affected by the number of pMad motifs in a stage-dependent manner. Double *in situ* hybridization against the *lacZ* driven by the pMad homotypic CRMs (A–D, red), the endogenous *dpp* gene (A′–C′, green) or the endogenous *tin* gene (D′, green). Schematic representations (A′″–D′″) indicate the CRM composition, where purple triangles depict pMad sites with indicated spacing (bp) between two adjacent sites. Amnioserosa activity is lost when the number of pMad sites is reduced at stage 14, while it remains unaffected at stage 11 when higher levels of pMad are present (A″–D″). Amnioserosa lacZ expression (D″, white arrow) does not overlap the heart (D″, green arrow) at stage 14. Embryos at stage 11 and 14 are lateral and dorsal views, respectively, with anterior to the left.
(PDF)

Figure S4 CRM activity in the amnioserosa is not affected by changes in the spacing and orientation of motifs. Double *in situ* hybridization against the *lacZ* reporter gene driven by the synthetic CRMs (A–I, red) and the endogenous *dpp* gene (A′–I′, green). Schematic representations (A′″–I′″) indicate CRM composition, where triangles (pMad – purple, Tin – green) depict the number and orientation of sites. Spacing between adjacent TF motifs (below) and pMad sites (above) is indicated. Expression in the amnioserosa is indicated with the arrow (A″–I″). All embryos are stage 13/14, shown in dorsal view with anterior to the left, except pMad-Tin A8 which is lateral.
(PDF)

Figure S5 Activity of two CRMs with the same pMad-Tin motif arrangement, but with a different spacer sequence, as used in Figure 3. Double *in situ* hybridization against the *lacZ* reporter gene driven by the synthetic CRMs (A,B, red) and the endogenous *dpp* gene (A′,B′, green). (A″,B″) CRM activity in midgut visceral mesoderm (VM) is indicated with arrowheads (A″,B″). Embryos are dorsally oriented, with anterior to the left, stage 13/14. (A′″,B′″) CRM composition, where triangles (pMad – purple, Tin – green) depict the number and orientation of sites. Spacing between adjacent pMad-Tin sites (below) and pMad-pMad sites (above) is indicated, with light grey bars between the triangles representing different spacer sequence than used in Figure 3.
(PDF)

Figure S6 Quantifying activity for heterotypic CRMs in the heart and validation of VM expressivity scoring. (A) Automated image analysis protocol for quantifying CRM expression in heart tissue. The expression pattern of the endogenous *tin* gene was digitized to create a mask of the tissue of interest. Comparing the mask with CRM activity enabled rapid and reliable scoring of both penetrance and expressivity, based on four heart regions (described in Materials and Methods). Errors in penetrance and expressivity were estimated as described in Supplemental Methods (Text S1). (B) Comparison of measured penetrance from all embryos (red bars) and from the subset of embryos selected for expressivity quantification (yellow bars).
(PDF)

Figure S7 Outline of the biophysical model used to model CRM activity. (A) Schematic of fractional occupancy model. For each CRM different possible cooperative TF configurations were identified. For each motif, the binding probability was adjusted by the binding weight (third panel) that represents the effect of binding site separation and orientation. Parameter fitting was performed by minimizing the residual error to both the penetrance and expressivity. (B) Example of an empty and bound binding site with corresponding weights as used in the model (see “Modeling CRM activity” ” in Supplemental Methods (Text S1)). (C) Schematic view of two different TFs binding to adjacent binding sites. The weights used in the fractional occupancy model for each state are shown. (D) Enumeration of the possible binding configurations of the 1× pMad-Tin CRM (left) and pMad-Tin-pMad CRM (center and right). The unbound state is given weight one. *a*_0_ and *b_0_* are the independent binding weights of pMad and Tin respectively, whilst α and *β* represent increased weight due to Tin-pMad and pMad-Tin-pMad cooperative interactions. Dashed ellipses denote cooperative TF interactions. The left column corresponds to the schematic case outlined in (C). For the pMad-Tin-pMad CRM, the center table only includes cooperative interactions between independent pairs of pMad-Tin, whereas the right table also includes potential higher-order TF interactions. (E) Schematic view of the binding function that describes how binding site orientation and separation alters the strength of cooperative TF interactions. The transcription factor binding domains are assumed to be spherical and overlapping (red bar).
(PDF)

Figure S8 Model testing and verification in the VM and heart. (A) Fitting of model to measured penetrance and expressivity for heterotypic 6× CRMs in the VM where only pMad-Tin cooperative interactions are considered (gray bars). (B) The quality of prediction (residual error = sum of the squares of the difference between theory and experiment, where a low value represents the best performance) of the models with different levels of TF cooperativity for the short pMad-Tin CRMs in VM: “pMad-Tin only” is a model where only pairs of cooperatively interacting TFs are considered; “with pMad-Tin-pMad” is the model that also includes higher order TF cooperative interactions. (C) Model fitting for ‘leave-two-out’ cross-validation, showing the fraction of predicted fits that were within a given tolerance (red curve), where tolerance is defined as the difference between model prediction and experimental values for the removed two CRMs. Black dashed line represents expected result for random fitting. (D) CRM activity for the Tin-pMad-Tin heterotypic CRM. Double *in situ* hybridization against the *lacZ* reporter gene driven by the synthetic CRMs (D, red) and the endogenous *dpp* gene (D′, green), where arrowheads indicate expression in the midgut visceral mesoderm. Embryo is dorsally oriented, with anterior to the left, stage 14. (D″) CRM composition, where triangles (pMad – purple, Tin – green) depict the number and orientation of sites. (E) Fitting of model to measured penetrance and expressivity for heterotypic 6× CRMs in the heart with only pMad-Tin cooperative interactions. Also shown are the model predictions for the reduced pMad-Tin heterotypic CRMs (black bars). (F) Fitting of model to measured penetrance and expressivity for heterotypic CRMs in the heart where pMad-Tin-pMad cooperative interactions are assumed to be the minimal TF configuration along with the model predictions for the reduced pMad-Tin heterotypic CRMs (black bars). In all panels, gray bars denote theoretical fits, black bars denote model predictions, red bars correspond to penetrance measurements, and blue bars correspond to expressivity measurements.
(PDF)

Figure S9 Structural model of interactions between pMad and Tin DNA binding domains on DNA with different motif spacing. Homology modeling of crystal structure data from TFs with homologous DNA binding domains, suggesting that protein interactions can only occur between DNA binding domains (DBDs) of pMad (purple) and Tin (green) when the motifs are in configurations that match our experimental results. Changing the orientation and spacing between the binding sites breaks these interactions, supporting the models predictions.
(PDF)

Table S1 Motif instance used for each TF, including a comparison to all known motifs for other TFs. Each row shows the original PWM as a logo (PWM ChIP data column) that was used to derive the motif instance cloned in the synthetic CRMs (Motif Instance Cloned in CRMs column). Note, this PWM was enriched in ChIP data for that factor, in all cases except for Pnt. As there was no ChIP data available for Pnt, a PWM computed based on footprints published in [1], [2] was used (see Supplemental Methods (Text S1)). . The “Motif Matches” column lists all matches with a p-value<1e-3 returned by TOMTOM [3] when searching ‘All *Drosophila*’ databases with the cloned motifs (MEME web site version 4.9.0 as of May 22, 2013, with Freq _A/T_ = 0.3, Freq_G/C_ = 0.2 and other parameters set to default values). The p-value returned by TOMTOM is indicated in brackets. A representative logo from the FlyFactorSurvey [4] database is given in the fifth column (model name in FlyFactorSurvey: bin_FlyReg_FBgn0045759, Mad_FlyReg_FBgn0011648, tin_FlyReg_FBgn0004110, twi_FlyReg_FBgn0003900, pnr_SANGER_5_FBgn0003117, Doc2_SANGER_5_FBgn0035956, pan_FlyReg_FBgn0085432, pnt_SANGER_5_FBgn0003118, Bap_Cell_FBgn0004862). The last column shows an alignment between the cloned motif (from column three, shown in color above) and the best fit derived from the FlyFactorSurvey PWM (shown in black below – see methods). Positions with low information content (overall or maximum relative entropy in column ≤0.6) are indicated with lower case. In the aligned regions, a ‘|’ denotes matching bases while a ‘.’ denotes mismatches. Besides the model that we previously published (shown in the PWM ChIP data column), we could not find an alternative PWM for *Drosophila* Mef2. The models used in this study generally match the most recently published models from bacterial-one-hybrid data (available in FlyFactorSurvey) or other sources including SELEX and DNaseI data. In most cases, motif mismatches occur at positions of low information content. Similarly, unaligned positions tend to be of lower relevance for the binding specificity. As expected, motifs from TFs known to compete for similar sites match the cloned motifs; for example the motif for Slp1/2 and Bin, similarly the motif for Brk and pMad. Both Slp and Brk are repressors known to restrict the spatial boundaries of expression, while Bin and pMad function as activators. At the time when we initiated this study, there was no PWMs available for Doc and Bap. The motif instance used for both factors contains a small core that overlaps the newly available bacterial-one-hybrid motifs, while the rest of the motifs diverge. The Doc motif has strong similarity to motifs of other factors (Zelda, Sna, l(1)sc, Da) and gave limited activity. The cloned Bap motif gave no activity, despite the presence of the AGTG core. Similarly, the Mef2 motif, although matching the vertebrate Mef2 site and the characterized specificity of this TF, gave no activity.
(PDF)

Table S2 Sequences of the synthetic CRMs. Synthetic CRMs are built from TF motifs, which are color coded as follows: Bap, Bin, Doc, dTCF, Mef2, pMad, GATA, Pnt, Tin, Twi, and spacers between TF sites in black. Restriction enzyme sites used for cloning of synthetic CRMs into the vectors are shown in small caps and underlined on both ends of the given sequence (see Materials and Methods for more details on the cloning procedure).
(DOCX)

Table S3 Effect of genomic position on CRM activity. The activity of heterotypic pMad-Tin synthetic CRMs built from three pMad and three Tin sites (A – antisense, S – sense orientation of Tin site with indicated spacing from 2–8 bp between adjacent sites) was assessed using the phiC31 system (integrase line nos-phiC31; attP40 on chr2L (cytology 25C7) [5]) and by random transgenesis for some CRMs using *P*-element transposons. The CRM activity in different tissues is indicated as follows: the dorsal mesoderm (DM), amnioserosa (AS), visceral mesoderm (VM) and heart (H). NA = Not available. The pMad-Tin A8 fly line (random site X) is an outlier in all the examined tissues compared to other fly lines. The activity of pMad-Tin S6 (random site X) fly line was not observed in the VM compared to the integrase fly line. This may be because the *P*-element line was examined in embryos generated from heterozygous adults (as it was homozygous lethal). This is the only line like this that we obtained from all 22 examined fly lines.
(PDF)

Table S4 Measurement of CRM penetrance. CRM penetrance in the VM and heart for homotypic pMad and heterotypic pMad-Tin CRMs. Tissue: VM = visceral mesoderm, H = heart. Embryo #: number of embryos in each experiment (most experiments had two independent embryo collections and *in situ* hybridizations). CRM activity: number of embryos in each experiment with tissue-specific expression of the CRM. Penetrance: fraction of total embryos with tissue-specific CRM expression. pMad-Tin A2 1.P denotes the *P*-element insertion line (random site X). All other lines were created by phiC31-mediated integration.
(PDF)

Table S5 Measurement of CRM expressivity. CRM expressivity in the VM and heart tissues for homotypic pMad and heterotypic pMad-Tin CRMs. Dorsal view: number of embryos in each subset orientated dorsally. Expressivity: measured expressivity from all embryos in subset. Expressivity (dorsal only): measured expressivity in dorsally aligned embryos. Other sections and nomenclature as Table S4.
(PDF)

Table S6 Parameters fit by the model using the measured CRM penetrance and CRM data for the 6× pMad-Tin CRMs. Parameter values from model fitting as described in Modeling CRM activity. r_sense_ and r_antisense_ denote the length scale (in bp) of cooperative TF interactions for sense and antisense orientated Tin binding sites respectively. *q_1_* and *q_2_* denote the effective cooperative binding between pMad-Tin and pMad-Tin-pMad (or Tin-pMad-Tin) respectively as described in Supplemental Methods (Text S1) section ‘Modeling CRM activity,’ where VM and H denote visceral mesoderm and heart tissue respectively.
(PDF)

Text S1 Supplemental information. Contains a detailed description of the design of the synthetic elements, including the linker sequence, the crystal-structure modeling, and the thermodynamic modeling of CRM activity.
(DOCX)
