# Supplementary figures and images for: Subtle Changes in Motif Positioning Cause Tissue-Specific Effects on Robustness of an Enhancer's Activity
Source: PLoS Genet. 2014 Jan 2;10(1):e1004060. doi: 10.1371/journal.pgen.1004060 (PMC3879207; doi:10.1371/journal.pgen.1004060)

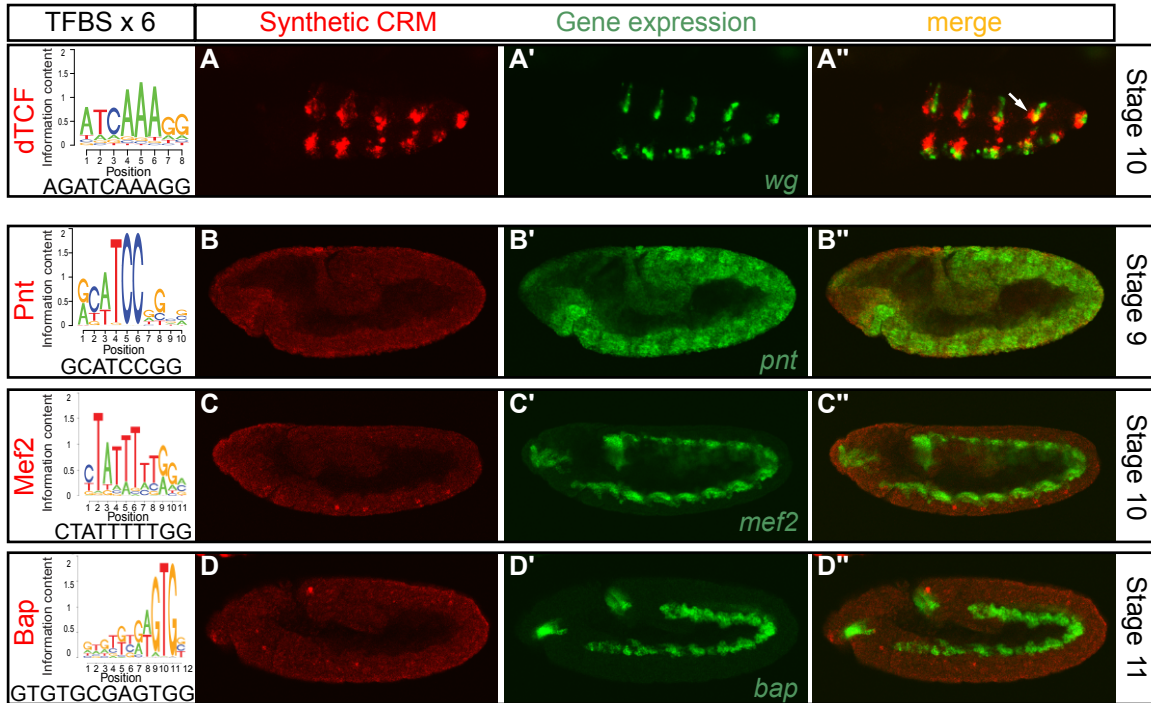

Supplement: Figure S1 — Spatio-temporal activity of homotypic synthetic CRMs. All homotypic synthetic CRMs contain optimized TFBS from in vivo occupancy data, represented by the sequence logo for each TF, repeated six times and separated with a spacer of 6 bp. Double fluorescent in situ hybridization of the lacZ reporter gene driven by the synthetic CRM (A–D, red) and the corresponding TF's endogenous gene (B′–D′, green), or wg in the case of dTCF (A′). The dTCF CRM drives expression in segmental groups of cells adjacent to wg expression (A, A″). Pnt (B, B″), Mef2 (C, C″) and Bap (D, D″) synthetic CRMs did not drive any specific expression. All embryos are laterally oriented with anterior to the left. (PDF) [file pgen.1004060.s001.pdf]

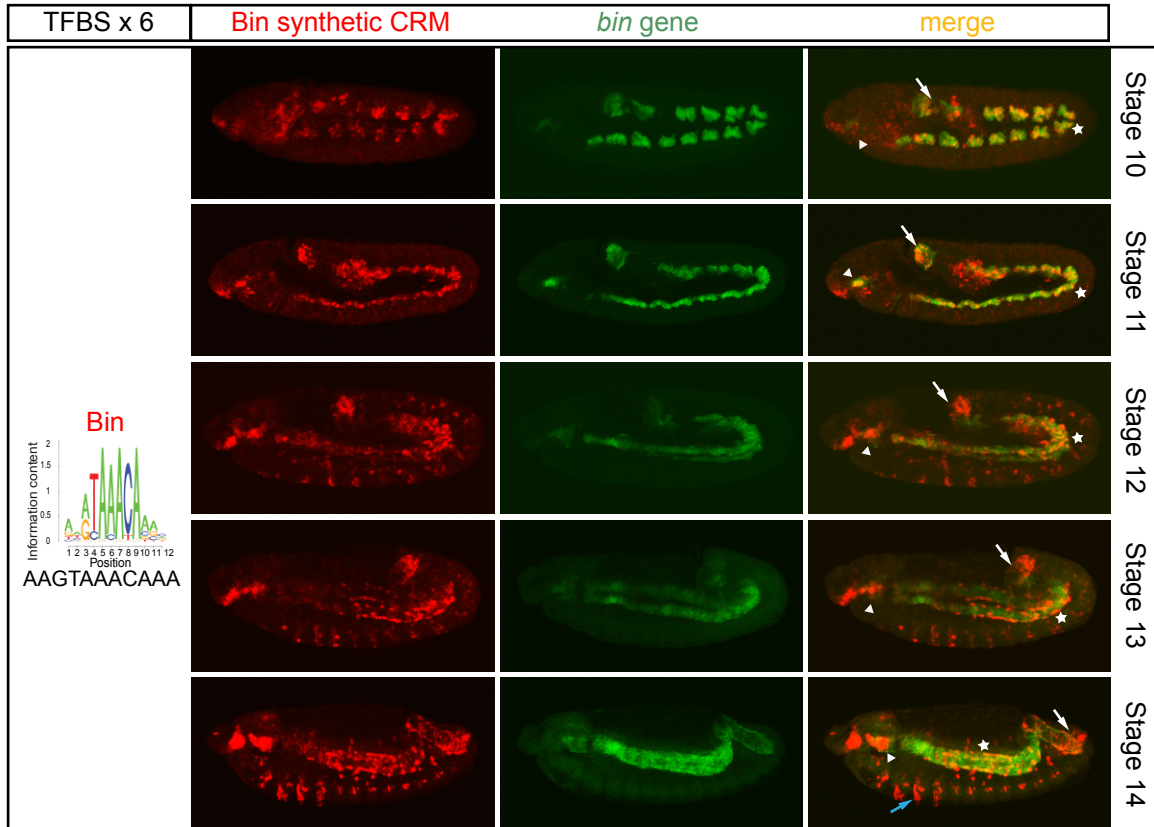

Supplement: Figure S2 — A synthetic CRM with six Bin motifs is sufficient to drive expression throughout the visceral mesoderm. The Bin motif (represented by the sequence logo) was multimerized six times, separated by a 6 bp, to generate the homotypic synthetic Bin CRM. Double in situ hybridization against the lacZ reporter gene driven by the synthetic CRM (red) and the endogenous bin gene (green), showing expression in the forgut (arrowhead), midgut (asterisk) and hindgut (arrow) visceral mesoderm as indicated by the colocalization with bin (yellow) during embryogenesis. Background plasmid activity is depicted with a blue arrow. All embryos are lateral views with anterior to the left. (PDF) [file pgen.1004060.s002.pdf]

**A**

## Image analysis of CRM activity in the heart

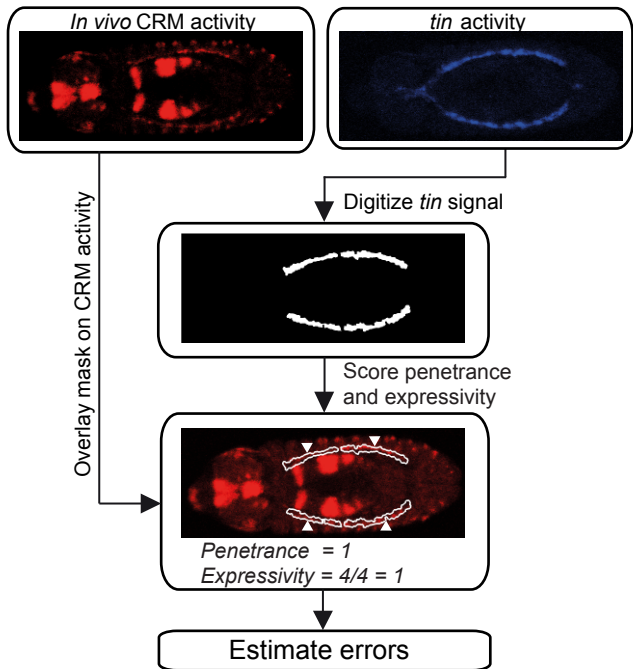**B**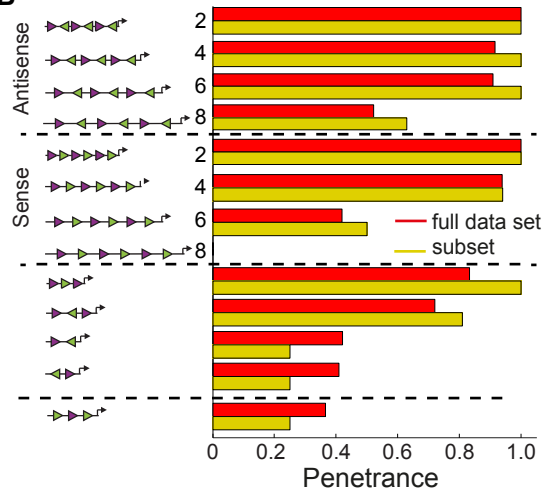

Supplement: Figure S6 — Quantifying activity for heterotypic CRMs in the heart and validation of VM expressivity scoring. (A) Automated image analysis protocol for quantifying CRM expression in heart tissue. The expression pattern of the endogenous tin gene was digitized to create a mask of the tissue of interest. Comparing the mask with CRM activity enabled rapid and reliable scoring of both penetrance and expressivity, based on four heart regions (described in Materials and Methods). Errors in penetrance and expressivity were estimated as described in Supplemental Methods (Text S1). (B) Comparison of measured penetrance from all embryos (red bars) and from the subset of embryos selected for expressivity quantification (yellow bars). (PDF) [file pgen.1004060.s006.pdf]

**A**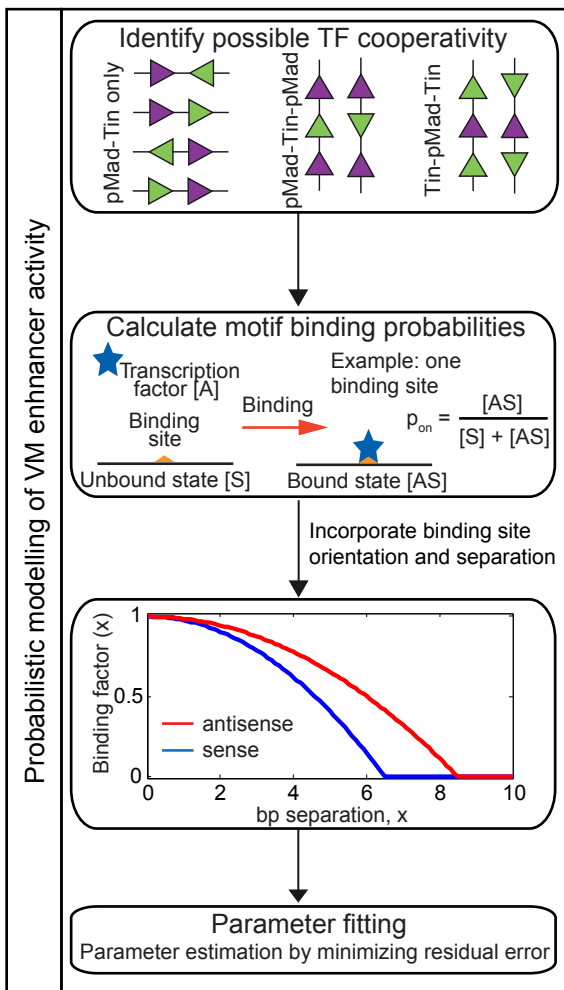**B**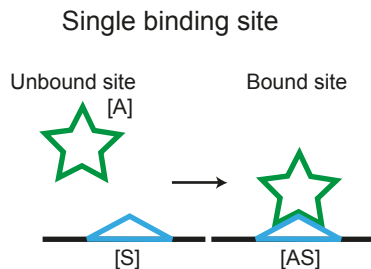**C**

### Two different binding sites

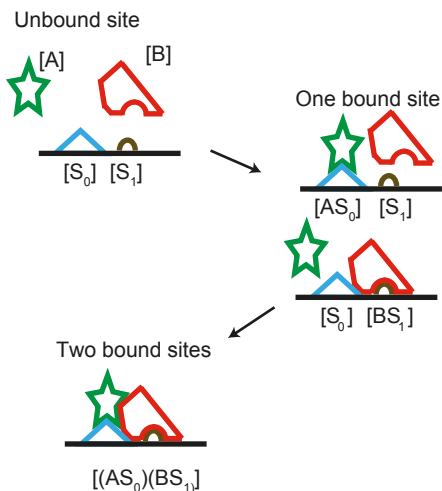**D**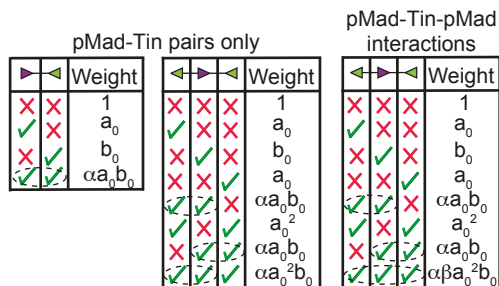**E**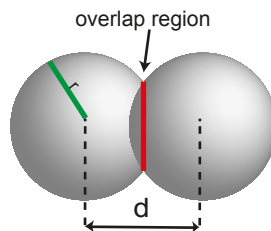

Supplement: Figure S7 — Outline of the biophysical model used to model CRM activity. (A) Schematic of fractional occupancy model. For each CRM different possible cooperative TF configurations were identified. For each motif, the binding probability was adjusted by the binding weight (third panel) that represents the effect of binding site separation and orientation. Parameter fitting was performed by minimizing the residual error to both the penetrance and expressivity. (B) Example of an empty and bound binding site with corresponding weights as used in the model (see “Modeling CRM activity” ” in Supplemental Methods (Text S1)). (C) Schematic view of two different TFs binding to adjacent binding sites. The weights used in the fractional occupancy model for each state are shown. (D) Enumeration of the possible binding configurations of the 1× pMad-Tin CRM (left) and pMad-Tin-pMad CRM (center and right). The unbound state is given weight one. a 0 and b0 are the independent binding weights of pMad and Tin respectively, whilst α and β represent increased weight due to Tin-pMad and pMad-Tin-pMad cooperative interactions. Dashed ellipses denote cooperative TF interactions. The left column corresponds to the schematic case outlined in (C). For the pMad-Tin-pMad CRM, the center table only includes cooperative interactions between independent pairs of pMad-Tin, whereas the right table also includes potential higher-order TF interactions. (E) Schematic view of the binding function that describes how binding site orientation and separation alters the strength of cooperative TF interactions. The transcription factor binding domains are assumed to be spherical and overlapping (red bar). (PDF) [file pgen.1004060.s007.pdf]

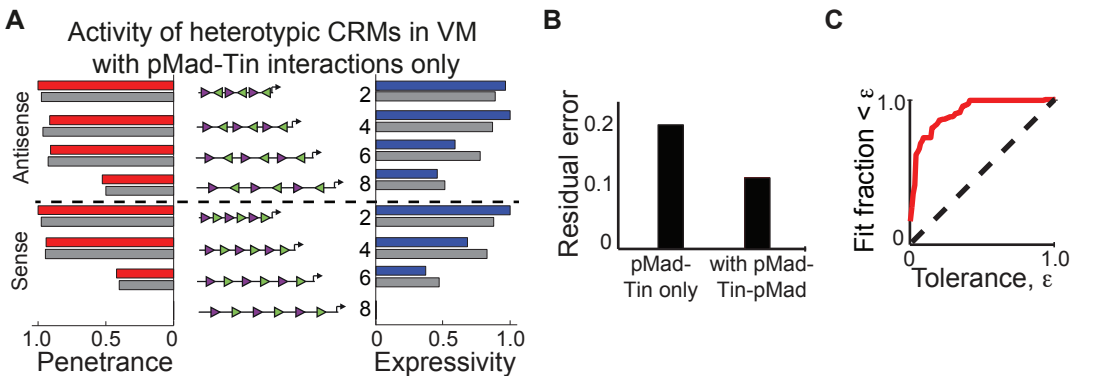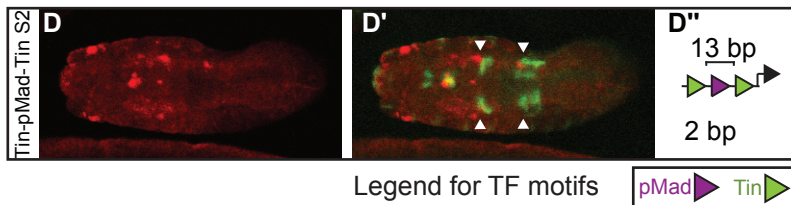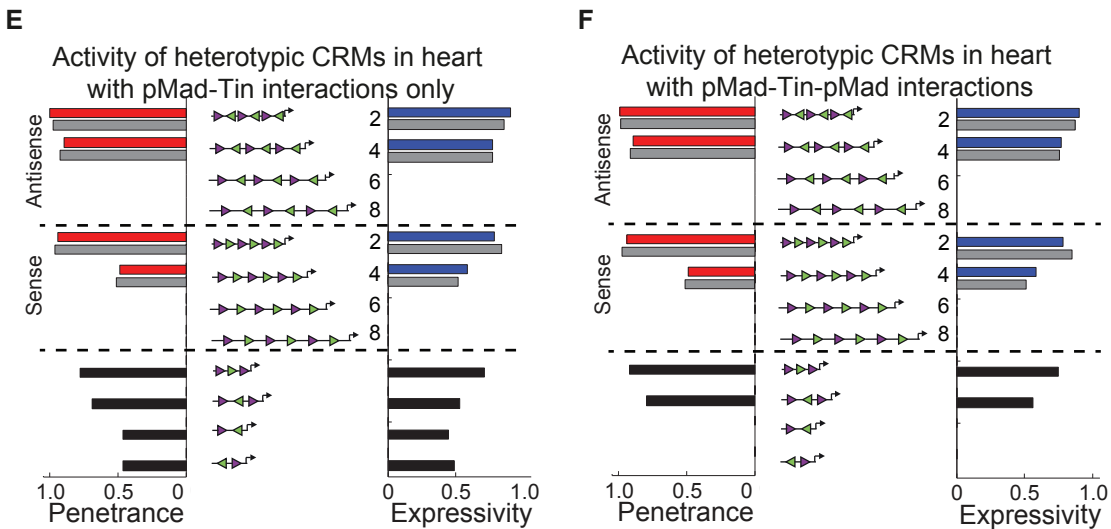

Supplement: Figure S8 — Model testing and verification in the VM and heart. (A) Fitting of model to measured penetrance and expressivity for heterotypic 6× CRMs in the VM where only pMad-Tin cooperative interactions are considered (gray bars). (B) The quality of prediction (residual error = sum of the squares of the difference between theory and experiment, where a low value represents the best performance) of the models with different levels of TF cooperativity for the short pMad-Tin CRMs in VM: “pMad-Tin only” is a model where only pairs of cooperatively interacting TFs are considered; “with pMad-Tin-pMad” is the model that also includes higher order TF cooperative interactions. (C) Model fitting for ‘leave-two-out’ cross-validation, showing the fraction of predicted fits that were within a given tolerance (red curve), where tolerance is defined as the difference between model prediction and experimental values for the removed two CRMs. Black dashed line represents expected result for random fitting. (D) CRM activity for the Tin-pMad-Tin heterotypic CRM. Double in situ hybridization against the lacZ reporter gene driven by the synthetic CRMs (D, red) and the endogenous dpp gene (D′, green), where arrowheads indicate expression in the midgut visceral mesoderm. Embryo is dorsally oriented, with anterior to the left, stage 14. (D″) CRM composition, where triangles (pMad – purple, Tin – green) depict the number and orientation of sites. (E) Fitting of model to measured penetrance and expressivity for heterotypic 6× CRMs in the heart with only pMad-Tin cooperative interactions. Also shown are the model predictions for the reduced pMad-Tin heterotypic CRMs (black bars). (F) Fitting of model to measured penetrance and expressivity for heterotypic CRMs in the heart where pMad-Tin-pMad cooperative interactions are assumed to be the minimal TF configuration along with the model predictions for the reduced pMad-Tin heterotypic CRMs (black bars). In all panels, gray bars denote theoretical fits, bla [file pgen.1004060.s008.pdf]

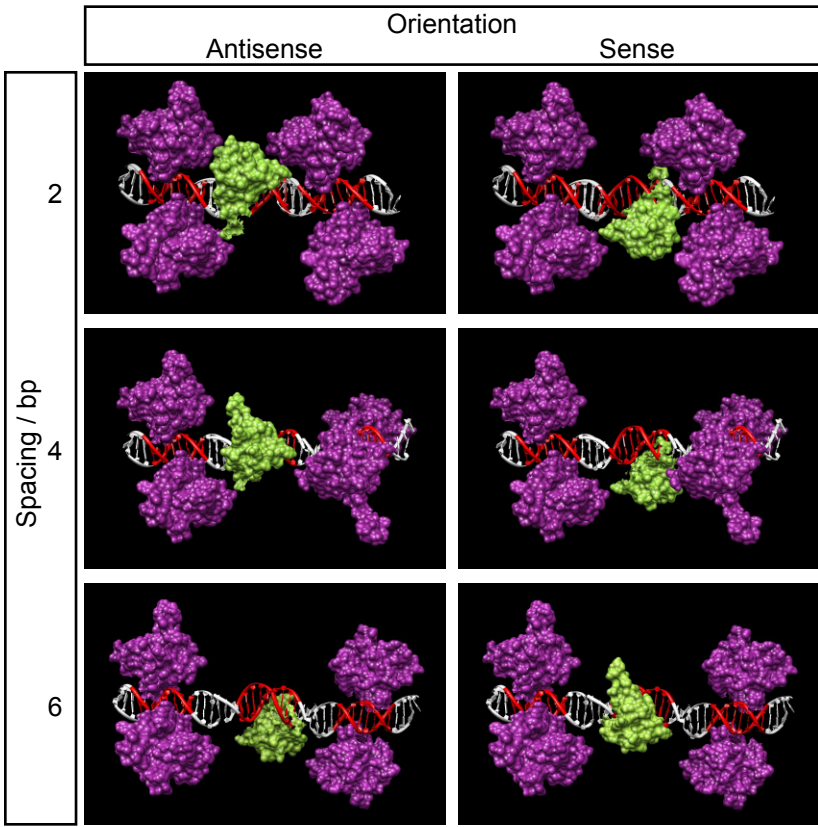

Supplement: Figure S9 — Structural model of interactions between pMad and Tin DNA binding domains on DNA with different motif spacing. Homology modeling of crystal structure data from TFs with homologous DNA binding domains, suggesting that protein interactions can only occur between DNA binding domains (DBDs) of pMad (purple) and Tin (green) when the motifs are in configurations that match our experimental results. Changing the orientation and spacing between the binding sites breaks these interactions, supporting the models predictions. (PDF) [file pgen.1004060.s009.pdf]
