# Supplementary material for: Subtle Changes in Motif Positioning Cause Tissue-Specific Effects on Robustness of an Enhancer's Activity
Source: PLoS Genet. 2014 Jan 2;10(1):e1004060. doi: 10.1371/journal.pgen.1004060 (PMC3879207; doi:10.1371/journal.pgen.1004060)
Supplement: Figure S3 — pMad homotypic CRM activity in the amnioserosa is affected by the number of pMad motifs in a stage-dependent manner. Double in situ hybridization against the lacZ driven by the pMad homotypic CRMs (A–D, red), the endogenous dpp gene (A′–C′, green) or the endogenous tin gene (D′, green). Schematic representations (A′″–D′″) indicate the CRM composition, where purple triangles depict pMad sites with indicated spacing (bp) between two adjacent sites. Amnioserosa activity is lost when the number of pMad sites is reduced at stage 14, while it remains unaffected at stage 11 when higher levels of pMad are present (A″–D″). Amnioserosa lacZ expression (D″, white arrow) does not overlap the heart (D″, green arrow) at stage 14. Embryos at stage 11 and 14 are lateral and dorsal views, respectively, with anterior to the left. (PDF) [file pgen.1004060.s003.pdf]

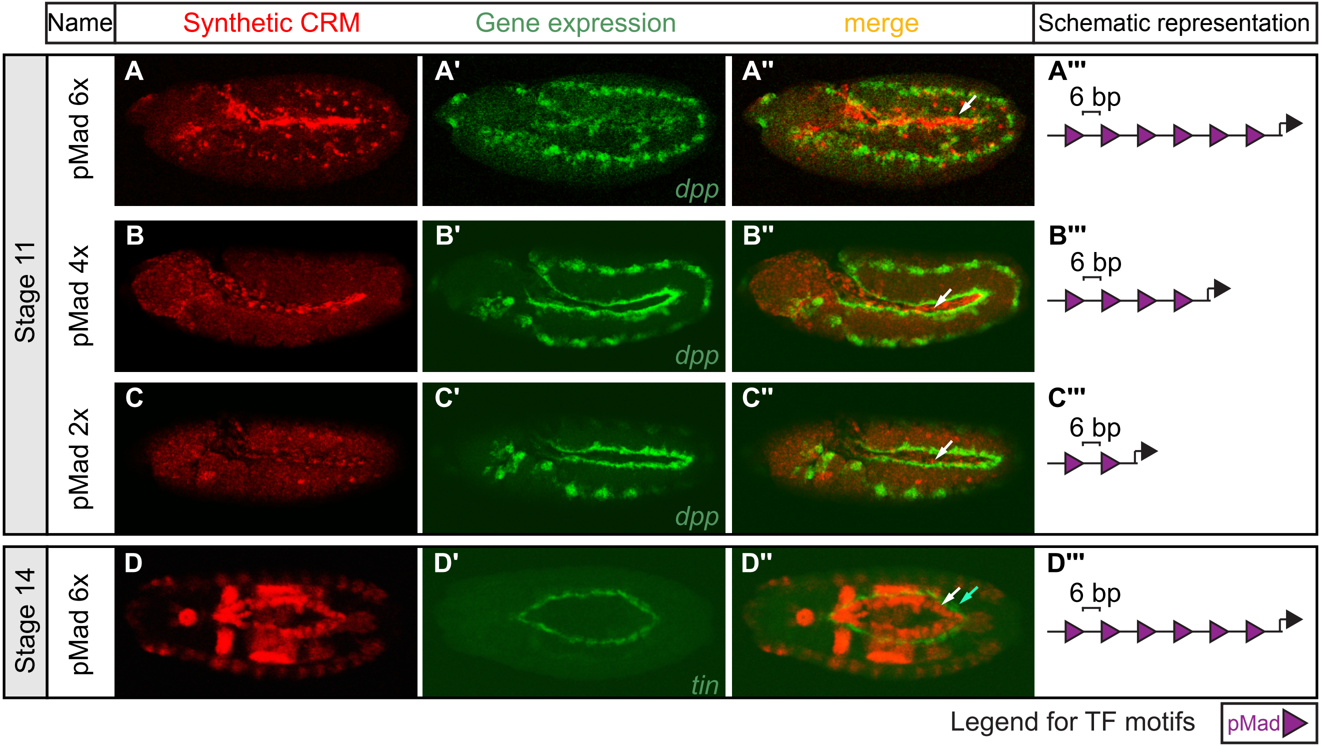

Name
Synthetic CRM
Gene expression
merge
Schematic representation
Stage 11
pMad 6x
A
A'
A''
A'''
dpp
6 bp
pMad 4x
B
B'
B''
B'''
dpp
6 bp
pMad 2x
C
C'
C''
C'''
dpp
6 bp
Stage 14
pMad 6x
D
D'
D''
D'''
tin
6 bp
Legend for TF motifs
pMad
