# Supplementary material for: Subtle Changes in Motif Positioning Cause Tissue-Specific Effects on Robustness of an Enhancer's Activity
Source: PLoS Genet. 2014 Jan 2;10(1):e1004060. doi: 10.1371/journal.pgen.1004060 (PMC3879207; doi:10.1371/journal.pgen.1004060)
Supplement: Figure S4 — CRM activity in the amnioserosa is not affected by changes in the spacing and orientation of motifs. Double in situ hybridization against the lacZ reporter gene driven by the synthetic CRMs (A–I, red) and the endogenous dpp gene (A′–I′, green). Schematic representations (A′″–I′″) indicate CRM composition, where triangles (pMad – purple, Tin – green) depict the number and orientation of sites. Spacing between adjacent TF motifs (below) and pMad sites (above) is indicated. Expression in the amnioserosa is indicated with the arrow (A″–I″). All embryos are stage 13/14, shown in dorsal view with anterior to the left, except pMad-Tin A8 which is lateral. (PDF) [file pgen.1004060.s004.pdf]

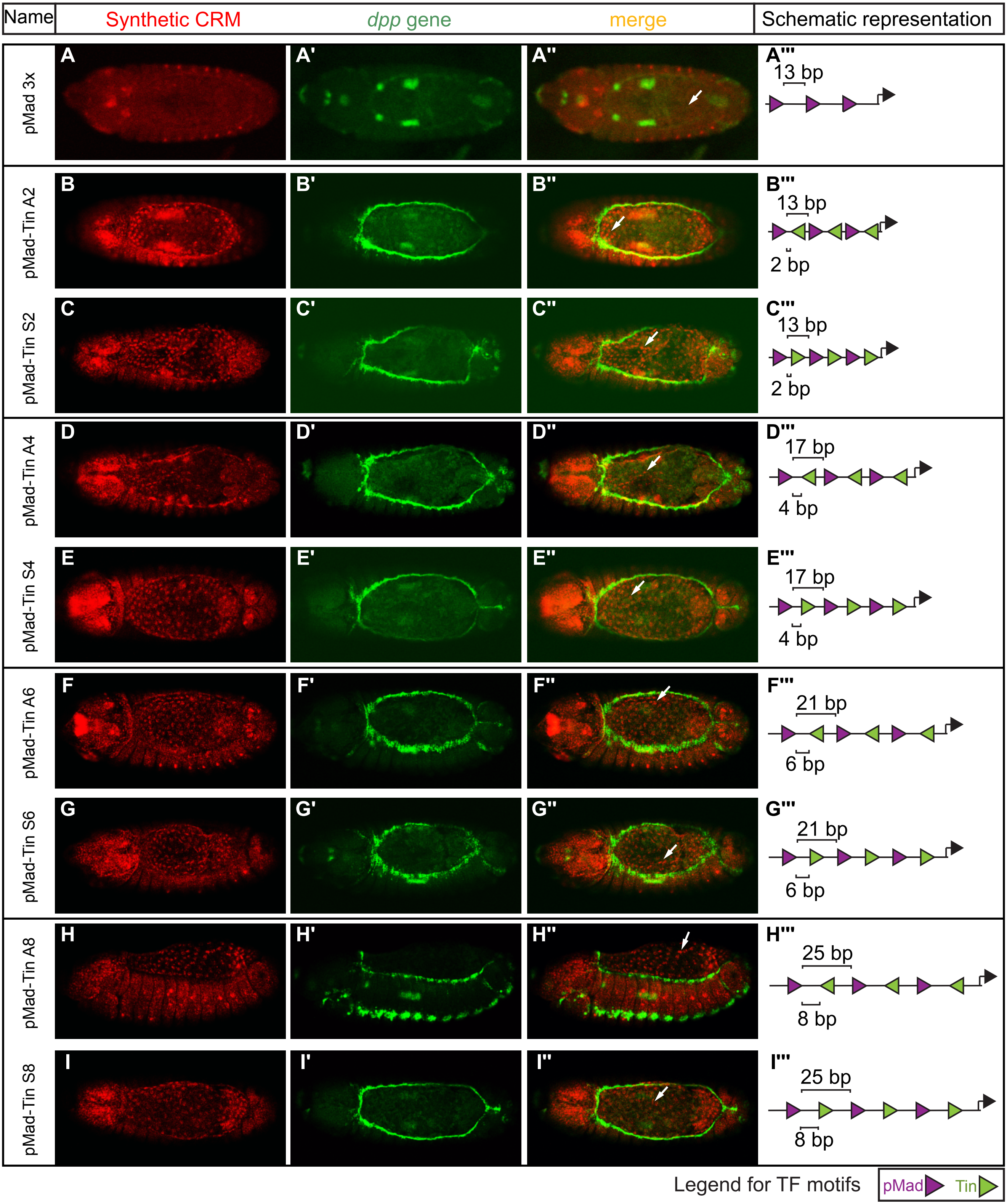

Name
Synthetic CRM
dpp gene
merge
Schematic representation
pMad 3x
A
A'
A''
A'''
13 bp
pMad-Tin A2
B
B'
B''
B'''
13 bp
2 bp
pMad-Tin S2
C
C'
C''
C'''
13 bp
2 bp
pMad-Tin A4
D
D'
D''
D'''
17 bp
4 bp
pMad-Tin S4
E
E'
E''
E'''
17 bp
4 bp
pMad-Tin A6
F
F'
F''
F'''
21 bp
6 bp
pMad-Tin S6
G
G'
G''
G'''
21 bp
6 bp
pMad-Tin A8
H
H'
H''
H'''
25 bp
8 bp
pMad-Tin S8
I
I'
I''
I'''
25 bp
8 bp
Legend for TF motifs
pMad
Tin
