# Supplementary material for: Subtle Changes in Motif Positioning Cause Tissue-Specific Effects on Robustness of an Enhancer's Activity
Source: PLoS Genet. 2014 Jan 2;10(1):e1004060. doi: 10.1371/journal.pgen.1004060 (PMC3879207; doi:10.1371/journal.pgen.1004060)
Supplement: Figure S5 — Activity of two CRMs with the same pMad-Tin motif arrangement, but with a different spacer sequence, as used in Figure 3. Double in situ hybridization against the lacZ reporter gene driven by the synthetic CRMs (A,B, red) and the endogenous dpp gene (A′,B′, green). (A″,B″) CRM activity in midgut visceral mesoderm (VM) is indicated with arrowheads (A″,B″). Embryos are dorsally oriented, with anterior to the left, stage 13/14. (A′″,B′″) CRM composition, where triangles (pMad – purple, Tin – green) depict the number and orientation of sites. Spacing between adjacent pMad-Tin sites (below) and pMad-pMad sites (above) is indicated, with light grey bars between the triangles representing different spacer sequence than used in Figure 3. (PDF) [file pgen.1004060.s005.pdf]

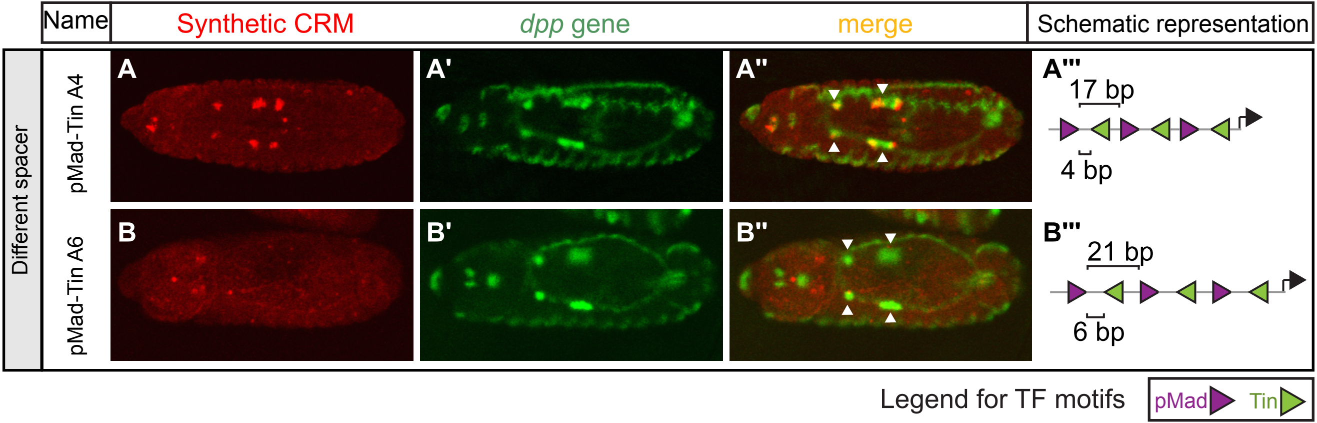

Name
Synthetic CRM
dpp gene
merge
Schematic representation
Different spacer
pMad-Tin A4
A
A'
A''
A'''
17 bp
4 bp
pMad-Tin A6
B
B'
B''
B'''
21 bp
6 bp
Legend for TF motifs
pMad
Tin
