# Supplementary material for: Subtle Changes in Motif Positioning Cause Tissue-Specific Effects on Robustness of an Enhancer's Activity
Source: PLoS Genet. 2014 Jan 2;10(1):e1004060. doi: 10.1371/journal.pgen.1004060 (PMC3879207; doi:10.1371/journal.pgen.1004060)
Supplement: Table S1 — Motif instance used for each TF, including a comparison to all known motifs for other TFs. Each row shows the original PWM as a logo (PWM ChIP data column) that was used to derive the motif instance cloned in the synthetic CRMs (Motif Instance Cloned in CRMs column). Note, this PWM was enriched in ChIP data for that factor, in all cases except for Pnt. As there was no ChIP data available for Pnt, a PWM computed based on footprints published in [1], [2] was used (see Supplemental Methods (Text S1)). . The “Motif Matches” column lists all matches with a p-value<1e-3 returned by TOMTOM [3] when searching ‘All Drosophila’ databases with the cloned motifs (MEME web site version 4.9.0 as of May 22, 2013, with Freq A/T = 0.3, FreqG/C = 0.2 and other parameters set to default values). The p-value returned by TOMTOM is indicated in brackets. A representative logo from the FlyFactorSurvey [4] database is given in the fifth column (model name in FlyFactorSurvey: bin_FlyReg_FBgn0045759, Mad_FlyReg_FBgn0011648, tin_FlyReg_FBgn0004110, twi_FlyReg_FBgn0003900, pnr_SANGER_5_FBgn0003117, Doc2_SANGER_5_FBgn0035956, pan_FlyReg_FBgn0085432, pnt_SANGER_5_FBgn0003118, Bap_Cell_FBgn0004862). The last column shows an alignment between the cloned motif (from column three, shown in color above) and the best fit derived from the FlyFactorSurvey PWM (shown in black below – see methods). Positions with low information content (overall or maximum relative entropy in column ≤0.6) are indicated with lower case. In the aligned regions, a ‘|’ denotes matching bases while a ‘.’ denotes mismatches. Besides the model that we previously published (shown in the PWM ChIP data column), we could not find an alternative PWM for Drosophila Mef2. The models used in this study generally match the most recently published models from bacterial-one-hybrid data (available in FlyFactorSurvey) or other sources including SELEX and DNaseI data. In most cases, motif mismatches occur at positions of low information conte [file pgen.1004060.s010.pdf]

Erceg, Table S1

Motif instance used for each TF, including a comparison to all known motifs for other TFs

TF	PWM ChIP data	Motif Instance Cloned in CRMs	Motif Matches (p<1e-3)	PWM FlyFactorSurvey	Motif Alignment
Bin	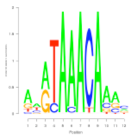	AAGTAAACAAA	slp2 (8e-6) ;<br>CG16899 (8.7e-5) ;<br>**bin (1e-4)** ;<br>slp1 (2e-4)	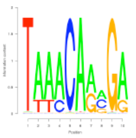	aagTAAACAaa<br>|||||||.<br>TAAACAacGA
pMad	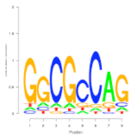	GGCGCCAG	brk (6e-6) ;<br>**pMad (9.4e-5)**	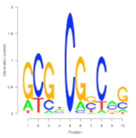	GGCGCCAG<br>|.||||.| GCGcCGsCcG
Tin	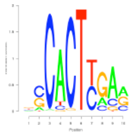	CCACTCGAG	**tin (1e-4) ;**<br>vnd (3.7e-4)	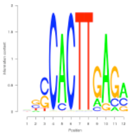	cCACTcGAg<br>|||||.||| CCACTTGAGa
Twist	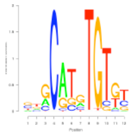	GCATGTGTGT	CG16778 (2.4e-5) ;<br>**twi (2e-4)** ;<br>da (3e-4) ;<br>ac_da (8e-4) ;<br>CG5953 (1e-3) ;<br>sc_da (1e-3)	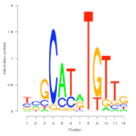	GCATgTGTGt<br>||||.|||.<br>GCATaTGTt
GATA	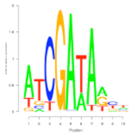	ATCGATA	BEAF-32 (7e-7) ;<br>Dref (4.7e-5)	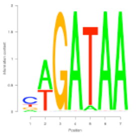	AtCGATA<br>.|||| aGATAA
Doc2	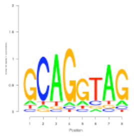	GCAGGTAG	vfl (2.5e-7) ;<br>wor (3e-4) ;<br>sna (4e-4) ;<br>l(1)sc_da (7e-4)	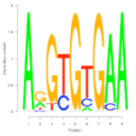	GCAGGtAG<br>||||..<br>AGGTGTGAA
dTcf	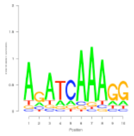	AGATCAAAGG	Hr51 (6.3e-5) ;<br>**dTcf (7.9e-5)**	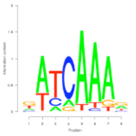	AgATCAAAGG<br>|||||| ATCAAA
Pnt	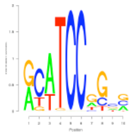	GCATCCGG	peb-F1-3 (5.1e-4) ;<br>aop (0.001)<br><br>N.B: **pnt (0.004)**	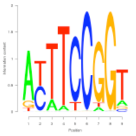	GCATCCgG<br>.|.||||| ACTTCCGGT
Mef2	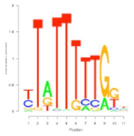	CTATTTTTGG	N.A.	N.A.	N.A.
Bap	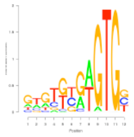	GTGTGCGAGTGG	klu (4e-4)	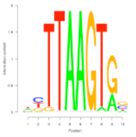	gtgtGcGaGTGg<br>...|||| TTAAGTG
