# Supplementary material for: Subtle Changes in Motif Positioning Cause Tissue-Specific Effects on Robustness of an Enhancer's Activity
Source: PLoS Genet. 2014 Jan 2;10(1):e1004060. doi: 10.1371/journal.pgen.1004060 (PMC3879207; doi:10.1371/journal.pgen.1004060)
Supplement: Table S2 — Sequences of the synthetic CRMs. Synthetic CRMs are built from TF motifs, which are color coded as follows: Bap, Bin, Doc, dTCF, Mef2, pMad, GATA, Pnt, Tin, Twi, and spacers between TF sites in black. Restriction enzyme sites used for cloning of synthetic CRMs into the vectors are shown in small caps and underlined on both ends of the given sequence (see Materials and Methods for more details on the cloning procedure). (DOCX) [file pgen.1004060.s011.docx]

**Erceg, Table S2**

**Homotypic synthetic CRMs**

>Bap synthetic CRM (120 bp)

gatctTCCATAGTGTGCGAGTGGTCCATAGTGTGCGAGTGGTCCATAGTGTGCGAGTGGTCCATAGTGTGCGAGTGGTCCATAGTGTGCGAGTGGTCCATAGTGTGCGAGTGGTCCATgc

>Bin synthetic CRM (115 bp)

gatctTCCATAAAGTAAACAAATCCATAAAGTAAACAAATCCATAAAGTAAACAAATCCATAAAGTAAACAAATCCATAAAGTAAACAAATCCATAAAGTAAACAAATCCATAgc

>Doc2 synthetic CRM (98 bp)

cgcgccTGACTAGCAGGTAGTGACTAGCAGGTAGTGACTAGCAGGTAGTGACTAGCAGGTAGTGACTAGCAGGTAGTGACTAGCAGGTAGTGACTAgg

>dTCF synthetic CRM (110 bp)

cgcgccaccctaagatcaaaggaccctaagatcaaaggaccctaagatcaaaggaccctaagatcaaaggaccctaagatcaaaggaccctaagatcaaaggaccctagg

>Mef2 synthetic CRM (109 bp)

gatctTCCATACTATTTTTGGTCCATACTATTTTTGGTCCATACTATTTTTGGTCCATACTATTTTTGGTCCATACTATTTTTGGTCCATACTATTTTTGGTCCATAgc

>pMad 6x synthetic CRM (98 bp)

cgcgccACGATGGGCGCCAGACGATGGGCGCCAGACGATGGGCGCCAGACGATGGGCGCCAGACGATGGGCGCCAGACGATGGGCGCCAGACGATGgg

>pMad 4x synthetic CRM (69 bp)

gatctACGATGGGCGCCAGACGATGGGCGCCAGACGATGGGCGCCAGACGATGGGCGCCAGACGATGgc

>pMad 3x synthetic CRM (72 bp)

gatcttaGGCGCCAGtaCTGTCCGAGtaGGCGCCAGtaCTGTCCGAGtaGGCGCCAGtaCTGTCCGAGtagc

>pMad 2x synthetic CRM (41 bp)

gatctACGATGGGCGCCAGACGATGGGCGCCAGACGATGgc

>GATA synthetic CRM (92 bp)

cgcgccGACTCTATCGATAGACTCTATCGATAGACTCTATCGATAGACTCTATCGATAGACTCTATCGATAGACTCTATCGATAGACTCTgg

>Pnt synthetic CRM (98 bp)

cgcgccAGTACCGCATCCGGAGTACCGCATCCGGAGTACCGCATCCGGAGTACCGCATCCGGAGTACCGCATCCGGAGTACCGCATCCGGAGTACCgg

>Tin 6x synthetic CRM (103 bp)

gatctTCCATACCACTCGAGTCCATACCACTCGAGTCCATACCACTCGAGTCCATACCACTCGAGTCCATACCACTCGAGTCCATACCACTCGAGTCCATAgc

>Tin 3x synthetic CRM (72 bp)

gatcttaAGTACTAGtaCCACTCGAGtaAGTACTAGtaCCACTCGAGtaAGTACTAGtaCCACTCGAGtagc

>Twi synthetic CRM (109 bp)

gatctTCCATAGCATGTGTGTTCCATAGCATGTGTGTTCCATAGCATGTGTGTTCCATAGCATGTGTGTTCCATAGCATGTGTGTTCCATAGCATGTGTGTTCCATAgc

**Heterotypic synthetic CRMs**

>Doc-Tin A2 synthetic CRM (72 bp)

gatcttaGCAGGTAGtaCTCGAGTGGtaGCAGGTAGtaCTCGAGTGGtaGCAGGTAGtaCTCGAGTGGtagc

>Doc-Tin S2 synthetic CRM (72 bp)

gatcttaGCAGGTAGtaCCACTCGAGtaGCAGGTAGtaCCACTCGAGtaGCAGGTAGtaCCACTCGAGtagc

>Doc-Tin A4 synthetic CRM (86 bp)

gatcttataGCAGGTAGtataCTCGAGTGGtataGCAGGTAGtataCTCGAGTGGtataGCAGGTAGtataCTCGAGTGGtatagc

>Doc-Tin S4 synthetic CRM (86 bp)

gatctgctaGCAGGTAGgctaCCACTCGAGgctaGCAGGTAGgctaCCACTCGAGgctaGCAGGTAGgctaCCACTCGAGgctagc

>Doc-Tin A6 synthetic CRM (100 bp)

gatctagtataGCAGGTAGagtataCTCGAGTGGagtataGCAGGTAGagtataCTCGAGTGGagtataGCAGGTAGagtataCTCGAGTGGagtatagc

>Doc-Tin S6 synthetic CRM (100 bp)

gatctggtataGCAGGTAGggtataCCACTCGAGggtataGCAGGTAGggtataCCACTCGAGggtataGCAGGTAGggtataCCACTCGAGggtatagc

>Doc-Tin S8 synthetic CRM (114 bp)

gatctggtatactGCAGGTAGggtatactCCACTCGAGggtatactGCAGGTAGggtatactCCACTCGAGggtatactGCAGGTAGggtatactCCACTCGAGggtatactgc

>dTCF-Tin A2 synthetic CRM (78 bp)

gatcttaAGATCAAAGGtaCTCGAGTGGtaAGATCAAAGGtaCTCGAGTGGtaAGATCAAAGGtaCTCGAGTGGtagc

>dTCF-Tin S2 synthetic CRM (78 bp)

gatcttaAGATCAAAGGtaCCACTCGAGtaAGATCAAAGGtaCCACTCGAGtaAGATCAAAGGtaCCACTCGAGtagc

>dTCF-Tin A4 synthetic CRM (92 bp)

gatctgtacAGATCAAAGGgtacCTCGAGTGGgtacAGATCAAAGGgtacCTCGAGTGGgtacAGATCAAAGGgtacCTCGAGTGGgtacgc

>dTCF-Tin S4 synthetic CRM (92 bp)

gatctgtacAGATCAAAGGgtacCCACTCGAGgtacAGATCAAAGGgtacCCACTCGAGgtacAGATCAAAGGgtacCCACTCGAGgtacgc

>dTCF-Tin A6 synthetic CRM (106 bp)

gatctgtacgtAGATCAAAGGgtacgtCTCGAGTGGgtacgtAGATCAAAGGgtacgtCTCGAGTGGgtacgtAGATCAAAGGgtacgtCTCGAGTGGgtacgtgc

>dTCF-Tin S6 synthetic CRM (106 bp)

gatctgtacgtAGATCAAAGGgtacgtCCACTCGAGgtacgtAGATCAAAGGgtacgtCCACTCGAGgtacgtAGATCAAAGGgtacgtCCACTCGAGgtacgtgc

>dTCF-Tin A8 synthetic CRM (120 bp)

gatcttgtacaccAGATCAAAGGtgtacaccCTCGAGTGGtgtacaccAGATCAAAGGtgtacaccCTCGAGTGGtgtacaccAGATCAAAGGtgtacaccCTCGAGTGGtgtacaccgc

>dTCF-Tin S8 synthetic CRM (120 bp)

gatctgtatactcAGATCAAAGGgtatactcCCACTCGAGgtatactcAGATCAAAGGgtatactcCCACTCGAGgtatactcAGATCAAAGGgtatactcCCACTCGAGgtatactcgc

>dTCF-Tin A10 synthetic CRM (119 bp)

gtacgtAGATCAAAGGtacagtacgtCTCGAGTGGtacagtacgtAGATCAAAGGtacagtacgtCTCGAGTGGtacagtacgtAGATCAAAGGtacagtacgtCTCGAGTGGtacagt

>pMad-Tin A2 synthetic CRM (72 bp)

gatcttaGGCGCCAGtaCTCGAGTGGtaGGCGCCAGtaCTCGAGTGGtaGGCGCCAGtaCTCGAGTGGtagc

>pMad-Tin S2 synthetic CRM (72 bp)

gatcttaGGCGCCAGtaCCACTCGAGtaGGCGCCAGtaCCACTCGAGtaGGCGCCAGtaCCACTCGAGtagc

>pMad-Tin A4 synthetic CRM (86 bp)

gatcttataGGCGCCAGtataCTCGAGTGGtataGGCGCCAGtataCTCGAGTGGtataGGCGCCAGtataCTCGAGTGGtatagc

>pMad-Tin A4 synthetic CRM (control with different spacer) (86 bp)

gatctGGtaGGCGCCAGGGtaCTCGAGTGGGGtaGGCGCCAGGGtaCTCGAGTGGGGtaGGCGCCAGGGtaCTCGAGTGGGGtagc

>pMad-Tin S4 synthetic CRM (86 bp)

gatcttataGGCGCCAGtataCCACTCGAGtataGGCGCCAGtataCCACTCGAGtataGGCGCCAGtataCCACTCGAGtatagc

>pMad-Tin A6 synthetic CRM (100 bp)

gatctagtataGGCGCCAGagtataCTCGAGTGGagtataGGCGCCAGagtataCTCGAGTGGagtataGGCGCCAGagtataCTCGAGTGGagtatagc

>pMad-Tin A6 synthetic CRM (control with different spacer) (100 bp)

gatctGAGTACGGCGCCAGGAGTACCTCGAGTGGGAGTACGGCGCCAGGAGTACCTCGAGTGGGAGTACGGCGCCAGGAGTACCTCGAGTGGGAGTACgc

>pMad-Tin S6 synthetic CRM (100 bp)

gatctggtataGGCGCCAGggtataCCACTCGAGggtataGGCGCCAGggtataCCACTCGAGggtataGGCGCCAGggtataCCACTCGAGggtatagc

>pMad-Tin A8 synthetic CRM (114 bp)

gatctgagtatacGGCGCCAGgagtatacCTCGAGTGGgagtatacGGCGCCAGgagtatacCTCGAGTGGgagtatacGGCGCCAGgagtatacCTCGAGTGGgagtatacgc

>pMad-Tin S8 synthetic CRM (114 bp)

gatctgatggtacGGCGCCAGgatggtacCCACTCGAGgatggtacGGCGCCAGgatggtacCCACTCGAGgatggtacGGCGCCAGgatggtacCCACTCGAGgatggtacgc

>pMad-Tin-pMad S2 synthetic CRM (40 bp)

gatcttaGGCGCCAGtaCCACTCGAGtaGGCGCCAGtagc

>pMad-Tin-pMad A4 synthetic CRM (48 bp)

gatcttataGGCGCCAGtataCTCGAGTGGtataGGCGCCAGtatagc

>Tin-pMad-Tin S2 synthetic CRM (41 bp)

gatctTACCACTCGAGtaGGCGCCAGtaCCACTCGAGTAgc

>1x pMad-Tin A4 synthetic CRM (36 bp)

gatcttataGGCGCCAGtataCTCGAGTGGtatagc

>1x Tin-pMad A4 synthetic CRM (36 bp)

gatcttataCTCGAGTGGtataGGCGCCAGtatagc

>mutpMad-mutTin S2 synthetic CRM (72 bp)

gatcttaAGTACTAGtaCTGTCCGAGtaAGTACTAGtaCTGTCCGAGtaAGTACTAGtaCTGTCCGAGtagc

>GATA-Tin A2 synthetic CRM (72 bp)

gatctacATCGATAGacCTCGAGTGGacATCGATAGacCTCGAGTGGacATCGATAGacCTCGAGTGGacgc

>GATA-Tin S2 synthetic CRM (72 bp)

gatctgtATCGATAGgtCCACTCGAGgtATCGATAGgtCCACTCGAGgtATCGATAGgtCCACTCGAGgtgc

>GATA-Tin A4 synthetic CRM (86 bp)

gatctgtacATCGATAGgtacCTCGAGTGGgtacATCGATAGgtacCTCGAGTGGgtacATCGATAGgtacCTCGAGTGGgtacgc

>GATA-Tin S4 synthetic CRM (86 bp)

gatctgtacATCGATAGgtacCCACTCGAGgtacATCGATAGgtacCCACTCGAGgtacATCGATAGgtacCCACTCGAGgtacgc

>GATA-Tin A6 synthetic CRM (100 bp)

gatcttgtaccATCGATAGtgtaccCTCGAGTGGtgtaccATCGATAGtgtaccCTCGAGTGGtgtaccATCGATAGtgtaccCTCGAGTGGtgtaccgc

>GATA-Tin S6 synthetic CRM (100 bp)

gatctgagtacATCGATAGgagtacCCACTCGAGgagtacATCGATAGgagtacCCACTCGAGgagtacATCGATAGgagtacCCACTCGAGgagtacgc

>GATA-Tin A8 synthetic CRM (114 bp)

gatcttgtacaccATCGATAGtgtacaccCTCGAGTGGtgtacaccATCGATAGtgtacaccCTCGAGTGGtgtacaccATCGATAGtgtacaccCTCGAGTGGtgtacaccgc

>GATA-Tin S8 synthetic CRM (114 bp)

gatctgatggtacATCGATAGgatggtacCCACTCGAGgatggtacATCGATAGgatggtacCCACTCGAGgatggtacATCGATAGgatggtacCCACTCGAGgatggtacgc

>GATA-Tin A10 synthetic CRM (119 bp)

gtacagtacATCGATAGtgtacagtacCTCGAGTGGtgtacagtacATCGATAGtgtacagtacCTCGAGTGGtgtacagtacATCGATAGtgtacagtacCTCGAGTGGtgtacagta

>GATA-Tin S10 synthetic CRM (119 bp)

taccaacgtATCGATAGgtaccaacgtCCACTCGAGgtaccaacgtATCGATAGgtaccaacgtCCACTCGAGgtaccaacgtATCGATAGgtaccaacgtCCACTCGAGgtaccaacg

>GATA-Tin-long A4 synthetic CRM (96 bp)

gatctATCGTGCGATAAGGtaccCTCGAGTGGtaccATCGTGCGATAAGGtaccCTCGAGTGGtaccATCGTGCGATAAGGtaccCTCGAGTGGgc

>GATA-Tin-long S4 synthetic CRM (96 bp)

gatctATCGTCCGATAAGGgtacCCACTCGAGgtacATCGTCCGATAAGGgtacCCACTCGAGgtacATCGTCCGATAAGGgtacCCACTCGAGgc

>GATA-Tin-long A6 synthetic CRM (106 bp)

gatctATCGTGCGATAAGGtataccCTCGAGTGGtataccATCGTGCGATAAGGtataccCTCGAGTGGtataccATCGTGCGATAAGGtataccCTCGAGTGGgc

>GATA-Tin-long S6 synthetic CRM (106 bp)

gatctATCGTCCGATAAGGtataccCCACTCGAGtataccATCGTCCGATAAGGtataccCCACTCGAGtataccATCGTCCGATAAGGtataccCCACTCGAGgc

>GATA-Tin-long A8 synthetic CRM (116 bp)

gatctATCGTGCGATAAGGtgtacaccCTCGAGTGGtgtacaccATCGTGCGATAAGGtgtacaccCTCGAGTGGtgtacaccATCGTGCGATAAGGtgtacaccCTCGAGTGGgc

>GATA-Tin-long S8 synthetic CRM (116 bp)

gatctATCGTGCGATAAGGagtataccCCACTCGAGagtataccATCGTGCGATAAGGagtataccCCACTCGAGagtataccATCGTGCGATAAGGagtataccCCACTCGAGgc

>GATA-Tin-long S10 synthetic CRM (119 bp)

ATCGTCCGATAAGGgacgtataccCCACTCGAGgacgtataccATCGTCCGATAAGGgacgtataccCCACTCGAGgacgtataccATCGTCCGATAAGGgacgtataccCCACTCGAG
