# Supplementary material for: Subtle Changes in Motif Positioning Cause Tissue-Specific Effects on Robustness of an Enhancer's Activity
Source: PLoS Genet. 2014 Jan 2;10(1):e1004060. doi: 10.1371/journal.pgen.1004060 (PMC3879207; doi:10.1371/journal.pgen.1004060)
Supplement: Table S3 — Effect of genomic position on CRM activity. The activity of heterotypic pMad-Tin synthetic CRMs built from three pMad and three Tin sites (A – antisense, S – sense orientation of Tin site with indicated spacing from 2–8 bp between adjacent sites) was assessed using the phiC31 system (integrase line nos-phiC31; attP40 on chr2L (cytology 25C7) [5]) and by random transgenesis for some CRMs using P-element transposons. The CRM activity in different tissues is indicated as follows: the dorsal mesoderm (DM), amnioserosa (AS), visceral mesoderm (VM) and heart (H). NA = Not available. The pMad-Tin A8 fly line (random site X) is an outlier in all the examined tissues compared to other fly lines. The activity of pMad-Tin S6 (random site X) fly line was not observed in the VM compared to the integrase fly line. This may be because the P-element line was examined in embryos generated from heterozygous adults (as it was homozygous lethal). This is the only line like this that we obtained from all 22 examined fly lines. (PDF) [file pgen.1004060.s012.pdf]

Erceg, Table S3

Effect of genomic position on CRM activity

Synthetic CRM	CRM activity											
	Integrase line				Random site X				Random site Y			
	DM	AS	VM	H	DM	AS	VM	H	DM	AS	VM	H
pMad-Tin A2	✔	✔	✔	✘	✔	✔	✔	✔	NA	NA	NA	NA
pMad-Tin S2	✔	✔	✔	✔	✔	✔	✔	✔	✔	✔	✔	✘
pMad-Tin A4	✔	✔	✔	✔	✔	✔	✔	✔	✔	✔	✔	✔
pMad-Tin S4	✔	✔	✔	✔	✔	✔	✔	✘	✔	✔	✔	✘
pMad-Tin A6	✔	✔	✔	✘	✔	✔	✔	✘	✔	✔	✔	✘
pMad-Tin S6	✔	✔	✔	✘	✔	✔	✘	✘	NA	NA	NA	NA
pMad-Tin A8	✔	✔	✔	✘	✘	✘	✘	✘	✔	✔	✔	✘
pMad-Tin S8	✔	✔	✘	✘	✔	✔	✘	✘	✔	✔	✘	✘
