# Supplementary material for: Subtle Changes in Motif Positioning Cause Tissue-Specific Effects on Robustness of an Enhancer's Activity
Source: PLoS Genet. 2014 Jan 2;10(1):e1004060. doi: 10.1371/journal.pgen.1004060 (PMC3879207; doi:10.1371/journal.pgen.1004060)
Supplement: Table S4 — Measurement of CRM penetrance. CRM penetrance in the VM and heart for homotypic pMad and heterotypic pMad-Tin CRMs. Tissue: VM = visceral mesoderm, H = heart. Embryo #: number of embryos in each experiment (most experiments had two independent embryo collections and in situ hybridizations). CRM activity: number of embryos in each experiment with tissue-specific expression of the CRM. Penetrance: fraction of total embryos with tissue-specific CRM expression. pMad-Tin A2 1.P denotes the P-element insertion line (random site X). All other lines were created by phiC31-mediated integration. (PDF) [file pgen.1004060.s013.pdf]

Erceg, Table S4

Measurement of CRM penetrance

CRM	Tissue	Embryo #	CRM Activity	Penetrance
pMad-Tin A2	VM	85, 25	85, 25	1
pMad-Tin A2 1.P	H	39	39	1
pMad-Tin S2	VM	89, 29	89, 29	1
pMad-Tin S2	H	89, 29	86, 25	0.94
pMad-Tin A4	VM	150, 26	135, 26	0.91
pMad-Tin A4	H	150, 26	131, 26	0.89
pMad-Tin S4	VM	38, 28	37, 25	0.94
pMad-Tin S4	H	38, 28	24, 8	0.48
pMad-Tin A6	VM	51, 103	48, 92	0.91
pMad-Tin S6	VM	35, 39	16, 15	0.42
pMad-Tin A8	VM	54, 13	26, 9	0.52
pMad-Tin-pMad S2	VM	24, 126	11, 97	0.72
pMad-Tin-pMad A4	VM	56, 100	51, 79	0.83
1x pMad-Tin A4	VM	71, 62	28, 28	0.42
1x Tin-pMad A4	VM	19, 74	12, 26	0.41
Tin-pMad-Tin S2	VM	112	41	0.37
