# Supplementary material for: Subtle Changes in Motif Positioning Cause Tissue-Specific Effects on Robustness of an Enhancer's Activity
Source: PLoS Genet. 2014 Jan 2;10(1):e1004060. doi: 10.1371/journal.pgen.1004060 (PMC3879207; doi:10.1371/journal.pgen.1004060)
Supplement: Table S5 — Measurement of CRM expressivity. CRM expressivity in the VM and heart tissues for homotypic pMad and heterotypic pMad-Tin CRMs. Dorsal view: number of embryos in each subset orientated dorsally. Expressivity: measured expressivity from all embryos in subset. Expressivity (dorsal only): measured expressivity in dorsally aligned embryos. Other sections and nomenclature as Table S4. (PDF) [file pgen.1004060.s014.pdf]

Erceg, Table S5

Measurement of CRM expressivity

CRM	Tissue	Embryo #	Dorsal view	Expressivity	Expressivity (dorsal only)
pMad-Tin A2	VM	15	11	0.97 ± 0.13	0.95 ± 0.15
pMad-Tin A2 1.P	H	16	10	0.98 ± 0.06	0.98 ± 0.08
pMad-Tin S2	VM	16	11	1	1
pMad-Tin S2	H	16	11	0.78 ± 0.29	0.77 ± 0.31
pMad-Tin A4	VM	15	9	1	1
pMad-Tin A4	H	15	9	0.77 ± 0.25	0.72 ± 0.25
pMad-Tin S4	VM	16	8	0.68 ± 0.22	0.68 ± 0.19
pMad-Tin S4	H	16	8	0.58 ± 0.31	0.45 ± 0.27
pMad-Tin A6	VM	16	11	0.59 ± 0.27	0.55 ± 0.15
pMad-Tin S6	VM	4	2	0.38 ± 0.18	0.25
pMad-Tin A8	VM	16	8	0.46 ± 0.10	0.40 ± 0.14
pMad-Tin-pMad S2	VM	16	13	0.59 ± 0.21	0.61 ± 0.23
pMad-Tin-pMad A4	VM	16	11	0.69 ± 0.27	0.61 ± 0.25
1x pMad-Tin A4	VM	16	11	0.44 ± 0.13	0.44 ± 0.13
1x Tin-pMad A4	VM	16	8	0.25 ± 0.00	0.25 ± 0.00
Tin-pMad-Tin S2	VM	16	8	0.38 ± 0.14	0.38 ± 0.14
