# Supplementary material for: Subtle Changes in Motif Positioning Cause Tissue-Specific Effects on Robustness of an Enhancer's Activity
Source: PLoS Genet. 2014 Jan 2;10(1):e1004060. doi: 10.1371/journal.pgen.1004060 (PMC3879207; doi:10.1371/journal.pgen.1004060)
Supplement: Table S6 — Parameters fit by the model using the measured CRM penetrance and CRM data for the 6× pMad-Tin CRMs. Parameter values from model fitting as described in Modeling CRM activity. rsense and rantisense denote the length scale (in bp) of cooperative TF interactions for sense and antisense orientated Tin binding sites respectively. q1 and q2 denote the effective cooperative binding between pMad-Tin and pMad-Tin-pMad (or Tin-pMad-Tin) respectively as described in Supplemental Methods (Text S1) section ‘Modeling CRM activity,’ where VM and H denote visceral mesoderm and heart tissue respectively. (PDF) [file pgen.1004060.s015.pdf]

Erceg, Table S6

Parameters fit by the model using the measured CRM penetrance and CRM data for the 6x pMad-Tin CRMs

Tissue	Cooperative TF interactions	r_{sense}	$r_{antisense}$	q_1	q_2
VM	pMad-Tin only	6.2bp	8.4bp	0.60	n/a
VM	pMad-Tin and pMad-Tin-pMad	6.4bp	8.8bp	0.19	1.9
H	pMad-Tin only	4.2bp	5.7bp	0.50	n/a
H	pMad-Tin-pMad only	4.3bp	6.0bp	n/a	4.8
H	Tin-pMad-Tin only	4.6bp	6.0bp	n/a	2.5

.
