# Supplementary material for: Subtle Changes in Motif Positioning Cause Tissue-Specific Effects on Robustness of an Enhancer's Activity
Source: PLoS Genet. 2014 Jan 2;10(1):e1004060. doi: 10.1371/journal.pgen.1004060 (PMC3879207; doi:10.1371/journal.pgen.1004060)
Supplement: Text S1 — Supplemental information. Contains a detailed description of the design of the synthetic elements, including the linker sequence, the crystal-structure modeling, and the thermodynamic modeling of CRM activity. (DOCX) [file pgen.1004060.s016.docx]

Supplemental Information

**Subtle changes in motif positioning cause tissue-specific effects on robustness of an enhancer’s activity**

Jelena Erceg, Timothy E. Saunders, Charles Girardot, Damien P. Devos, Lars Hufnagel, and Eileen E. M. Furlong

**Overview of Supplemental Information**

Supplemental Figures

**Figure S1.** Spatio-temporal activity of homotypic synthetic CRMs

**Figure S2.**  A synthetic CRM with six Bin motifs is sufficient to drive expression throughout the visceral mesoderm

**Figure S3.**  pMad homotypic CRM activity in the amnioserosa is affected by the number of pMad motifs in a stage-dependent manner

**Figure S4.** CRM activity in the amnioserosa is not affected by changes in the spacing and orientation of motifs

**Figure S5.** Activity of two CRMs with the same pMad-Tin motif arrangement using a different spacer sequence as used in Figure 3

**Figure S6.** Quantifying activity for heterotypic CRMs in the heart and validation of VM expressivity scoring

**Figure S7.** Outline of the biophysical model used to model CRM activity

**Figure S8.** Model testing and verification in the VM and heart

**Figure S9.**  Structural model of interactions between pMad and Tin DNA binding domains on DNA with different motif spacing

Supplemental Tables

**Table S1.** Motif instance used for each TF, including a comparison to all known motifs for other TFs

**Table S2.** Sequences of the synthetic CRMs

**Table S3.** Effect of genomic position on CRM activity

**Table S4.** Measurement of CRM penetrance

**Table S5.** Measurement of CRM expressivity

**Table S6.** Parameters fit by the model using the measured CRM penetrance and CRM data for the 6x pMad-Tin CRMs

Supplemental Methods

Supplemental References

## Supplemental Methods

**Construction of a PWM for Pnt**

The model for Pnt was generated using published footprints in [1,2] using the procedure described in [6,7]. Briefly, footprints were supplemented with manually curated orthologous sequences from *D.yakuba, D.ananassae, D.pseudoobscura, D.simulans, D.mojavensis* and *D.virilis* (ungapped alignments only, extracted from UCSC BlastZ pairwise alignments [8]) to construct PWMs using the MEME algorithm [1,2].

**Design of a linker sequence for Twi, Mef2, Bap, Bin, Tin homotypic CRMs**

All possible enumerations of 4 bases in a 6-mer (4096 possibilities) were considered as potential linker sequences. For each 6-mer K and for each TF (Twi, Mef2, Bap, Bin, Tin), a test sequence corresponding to the construct "TFBS-linker-TFBS-linker-TFBS" was assembled. We call these 5 test sequences, the T_K_ sequences. First (step 1), we discarded any 6-mer where any of its T_K_ sequences contain a PWM match with a score >1 for Twi, Mef2, Bap, Bin or Tin PWM (ignoring the T_K_ sequence for the TF of the current PWM). Since we aim to minimize the match scores of the linker sequence, a PWM match to a T_K_ sequence is only considered if a minimum length "min_len" of the linker is included in the matched sequence (here a min_len of 4 was used *i.e.* half of the linker sequence + 1 base). Match scores were computed as implemented in the patser tool [9] considering a GC percentage of 40% and a pseudocount of 1.  Second (step 2), we discarded any 6-mer which sequence (*i.e.* not the corresponding T_K_ sequences) is exactly found in a list of known footprints (as downloaded from REDfly in June 2008).  Third (step 3), the 6-mer sequence (*i.e.* not the corresponding T_K_ sequences) of the remaining 6-mers is evaluated against each of the 86 PWM models (91 models available for *Drosophila* TFs, minus the ones already used at step 1) and the *best fitting score* (considering all possible 6-mer/PWM alignments, (note that the PWM positions that are not aligned to the 6-mer are ignored when calculating the score) is recorded. In addition, the T_K_ sequences of the remaining n-mers are scored against all 86 PWMs (91 models minus the ones already used at step 1) and PWM matches of score greater than (or equals to) 2 are recorded for further inspection (for convenience, we refer to this set of matches as S).  Finally, the 6-mer with the lower 'best fitting score' was selected (here TCCATA) amongst the remaining candidates.

**Design of a unique linker sequences for GATA, Doc2, dTCF, pMad, Pnt homotypic CRMs**

Although it was possible to find a common low-matching linker sequence for Twi, Mef2, Bap, Bin, Tin homotypic CRMs, this was not possible for the other TFs. A common linker in combination with the motif for GATA, Doc2, dTCF, pMad and Pnt had a too high score to known PWMs and therefore generated the risk of inadvertently generating a new TF binding site. To address this, we designed a linker sequence for each of Doc2, dTCF, pMad, and Pnt individually (which resulted in a less stringent first step as the T_K_ sequence set then contains a unique member).  To increase the stringency in the selection procedure, we used a varying min_len (starting with 1, increasing in steps of 1) and stopped as soon as the procedure reported a solution. In one situation (dTCF), we did not select the lowest 'best fitting score' but the second lowest after inspection of the PWM matches set S (produced at step 3).

**Design of a unique linker sequences for heterotypic CRMs**

As for GATA, Doc2, dTCF, pMad, Pnt homotypic CRMs, we designed a linker for each heterotypic CRM (*i.e.* for each TF pair, linker size and relative Tin orientation). A second version of the linker design procedure has been developed to accommodate for the varying linker lengths and the heterotypic nature of the TFs used. We describe below the modifications made to the linker design procedure: (1) For a particular n-mer, where n belongs to {2,4,6,8}, TF pair (TF_1_ and Tin) and relative orientation of the Tin TFBS (sense or antisense), T_K_ contains a unique sequence corresponding to "TFBS_1_-linker-TFBS_Tin_-linker-TFBS_1_" (where linker is the current n-mer, TFBS_1_ and TFBS_Tin_ are the best transcription factor binding sites for TF_1_ and Tin with TFBS_Tin_ possibly reversed-complemented if the orientation is ‘antisense’). (2) At filtering step one, the “min_len” parameter has been removed and the contribution of a linker to a PWM match is evaluated as follows (only matches with score > 2 were considered). For each PWM match, we compute (a) the “linker score in match”, which is the part of the match score imputable to the linker bases and (b) the “linker score proportion”, which is the “linker score in match” divided by the overall match score. Linker candidates (*i.e.* n-mers) which “linker score proportion” was more than 40% of the overall match score or which “linker score in match” was greater than 1 were discarded. (3) The second step was omitted.

In step three, the T_K_ sequence of each remaining n-mer is evaluated against each of the 91 PWM models (removing the ones already used at step 1). The “linker score in match” is then computed for each match that (1) has a score > 0 and (2) involves 1 or more base of the linker sequence. Finally, all positive “linker score in match” are summed and the linker that has the lowest “linker score in match” sum was selected for the CRM design.

**Visualization of protein-protein interactions using crystal structure data**

Homologous sequences were collected by PSI-BLAST. Hidden Markov Models were built and screened against similar models for all proteins of known structure using the HHsearch suite of programs [10]. Secondary structure and coiled-coil predictions were made by PSIPRED [11]. Full-atoms three-dimensional models with DNA binding site were built using Modeller [12] based on SMAD1-MH1 from *Mus musculus* (PDB code 3kmp) [13]­­ for pMad, and HoxA9 homeodomain from *Mus musculus* (1puf) [14] for Tin. Localizations of the proteins with varying motif spacing were built by overlapping the bases in the DNA of the models. Binding of two pMad DNA binding domains were allowed on one pMad binding sites, as the site has palindromic nature [15,16]. Visualizations of protein interaction on DNA elements with changing parameters of spacing and orientation were performed using UCSF Chimera [17].

**Modeling CRM activity**

Examining the activity of the pMad-Tin heterotypic CRMs suggests that there may be simple rules regarding the spacing and orientation of motifs that link motif organization to robust activity. The sharp transitions induced by changes to motif configurations (such as spacing and orientation) suggest that these rules involve cooperative interactions across multiple proteins bound to a CRM. To better explore the biophysical mechanisms and logical rules underlying CRM behavior, we made use of fractional site occupancy modeling (shown schematically in Figure S7). Fractional site occupancy models are a family of models that borrow from statistical physics to describe DNA-protein and protein-protein interactions as thermodynamic processes. Within these models, the probability that a CRM is active is a function of the relative probabilities, over all possible combination of binding events, that a sufficient number of TFBSs on the CRM are stably bound. An advantage of such models is that they are conceptually very simple but yet they can incorporate considerable complexity with comparatively few parameters. For an introduction to thermodynamic modeling of *cis* regulatory regions see [18].

The size of the state space, including all possible binding configurations, of fractional occupancy models increases exponentially with the number of sites. The description of even simple CRMs activities might require many, *a priori* unknown, parameters without any apparent functional relationships. To avoid this problem, we decided to analysis and compare a series of models with increasing complexity. Our aim was to obtain the simplest model that recapitulates the observed experimental data, to help to structure and identify important CRM configurations in a mathematically stringent way.

For heterotypic pMad-Tin CRMs there are eight different six-TF motif CRM architectures, giving a total of 16 data points (8 penetrance and 8 expressivity). The emphasis of the model was to provide a qualitative framework for understanding how cooperative TF interactions may explain these results. The model, as detailed below, incorporates binding site number, orientation and separation with at most four parameters.

**Underlying assumptions**

Before discussing the details of the model, the main assumptions are outlined.

1. Only cooperative TF binding to the CRM is important in explaining the variation in activity observed between CRMs with different motif configurations.

*Rationale*: The focus of the modeling is to better understand how cooperative TF binding affects tissue-specific activity using different CRM architectures. The downstream processes from transcription factor binding - such as cofactor recruitment - and additional mechanisms such as chromatin structure modifications are assumed to have equal affect on all CRMs and hence can be ignored.

2. The time taken for the enhancer to become active was the rate-limiting step

*Rationale*: This assumption is necessary for a thermodynamic model to be appropriate for analyzing the data. In effect, this assumption means that the TF binding to the CRM is the key process in determining CRM activity.

3. The different tissue-specific regions (PS3, PS7) are similar with respect to factors that affect the CRM activity (such as pMad concentration) and that the conditions (e.g. protein concentrations) are the same for all cells within a given region.

*Rationale*: Although there are no quantitative TF concentration measurements available, visualizing pMad and Tin protein levels by immunostaining does not indicate that there is major intra-tissue variation in protein levels. Furthermore, for CRMs with variation in activity, we typically observe that the expression pattern is spread across adjacent cells rather than a salt-and-pepper distribution of expressing cells. This suggests that for a given tissue, the region-to-region variability in relevant factors (such as TF concentration) is considerably greater than the intra-region (*i.e.* cell-to-cell) fluctuations. With this assumption, the model effectively finds the probability of a CRM in a region being active rather than calculating the probability of CRM activity in individual cells.

**Modeling details**

The general modeling approach is to assign each possible combination of TF binding events a weight, Figure S7. The probability of a CRM being active is the sum of the weights that correspond to CRM activity divided by the total weight of all possible states. For an introduction to the formulation of thermodynamic models when modeling protein binding and cooperativity see (Ay and Arnosti, 2011). There are a number of possible mechanisms for TF binding to the CRMs including TFs acting as pioneer factors. However, our focus is on understanding the *minimum* level of TF binding cooperativity required for CRM activity. Therefore, precise details of the TF binding mechanisms are not necessary in the model. Here, we consider the scenario where TF binding occurs during the appropriate stages of development. The relevant TF proteins are assumed to be present in sufficiently high concentration that the binding of TFs to the CRM is not the rate-limiting step (in line with Assumption 2 above). We have also confirmed that our results hold if we consider some TFs behaving as pioneer factors (data not shown).

In the main paper we analyze the six TFBS CRMs before using these results to predict the behavior of smaller CRMs. However, for ease of conceptual understanding we present here the model analysis starting from the simplest possible CRMs. First, an example of an enhancer with a single binding site is discussed. We then explore the specific CRMs architectures used in the experiments. For the main discussion below the focus is on modeling of the CRM activity in the VM. Later, we discuss how the model is modified to be appropriate for understanding CRM activity in the heart.

**One transcription factor binding site**

For a single TFBS there are two possible states, as shown in Figure S7B. The binding site is either empty, with weight [*S_0_*] or the TF is bound, with weight [A*S_0_*] (where *[A]* denotes the TF protein concentration). The total weight of different states, denoted by *Z,* is given by

*Z = [S_0_] +[AS_0_] = [S_0_](1+[A]/K_d_)* (1)

Under detailed balance *[AS_0_] = [S_0_][A]/K_d_* where *K_d_* is the disassociation constant. If the occupied state *[AS_0_]* corresponds to the enhancer being active (*on*), then the probability of expression is given by

*p_on_ = ([A]/K_d_)/(1+[A]/K_d_)* (2)

In this case, the probability of CRM activity is a function of a single parameter, *q_0_ = [A]/K_d_*. For very high *K_d_*, *q_0_* is small and the probability of the enhancer being on is small - *i.e.* TFs disassociate very quickly and readout is unlikely. For very low *K_d_* (*i.e.* once bound, the TF remains bound for a long period) p_on_≈1 and the CRM is active. We also see that for *[A]*≈0 the probability of expression goes to zero, as expected.

**One pMad and one Tin binding site**

We next consider the smallest experimentally measured CRM: one pMad and one Tin binding site. For this CRM there are four possible states (shown schematically in Figure S7C): (1) both sites are empty, with weight *[S_0_S_1_]* (where *[S_0_]* and *[S_1_]* correspond to the pMad and Tin binding sites respectively); (2) only the pMad binding site is occupied (by a TF concentration denoted by *[A]*) with weight *[(AS_0_)S_1_]*; (3) only the Tin binding site is occupied (by a TF with concentration denoted by *[B]*) with weight *[S_0_(BS_1_)]*; (4) both sites are occupied with weight *[(AS_0_)(BS_1_)]*. The total weight is now given by

*Z = [S_0_S_1_]+[(AS_0_)S_1_]+[S_0_(BS_1_)]+[(AS_0_)(BS_1_)]* (3)

As above, from detailed balance *[AS_0_] = [S_0_][A]/K_d_^A^* and *[BS_1_] =* [S_1_][B]/K_d_^B^ (where *K_d_^A^* and *K_d_^B^* are the disassociation constants for transcription factors *[A]* and *[B]* respectively). However, the weight of the final state *[(AS_0_)(BS_1_)]* depends on whether, as the experiments suggest, there is cooperativity between the TFs when binding to the CRM. To incorporate cooperativity, the weight of the state with two proteins bound is assumed to be enhanced by α (regardless of which TF bound first - with the detailed balance assumption only the state itself need be considered when calculating the weight). The weights of the different states are shown in Figure S7D, where the formation of a cooperatively bound pair of transcription factors is represented by the ellipse covering both sites. If pMad must be bound in the VM for CRM activity (a reasonable assumption, given the lack of VM signal in the Tin homotypic CRM) then the probability of the enhancer being active is given by

*p_on_ = (a_0_+αa_0_b_0_)/(1+a_0_+b_0_+αa_0_b_0_)* (4)

where *a_0_ = [A]/K_d_^A^* and *b_0_ = [B]/K_d_^B^*. Setting *α = 1* corresponds to independent binding between the different sites. *α>1* corresponds to cooperative binding and *α<1* corresponds to antagonistic binding.

From the pMad-Tin S8 CRM result, we expect *a_0_* and *b_0_* to be small since this CRM displays no tissue-specific activity. Therefore, taking the limits *α>>1/a_0_* and*, α>>1/b_0_* (since individual binding of TF is unlikely) we find *p_on_ ≈ q_1_/(1+q_1_),* where *q_1_ =αa_0_b_0_*. *q_1_* corresponds to the weight of a state where a single pair of pMad and Tin cooperatively bind. Within our approximations, a single parameter describes the probability of the CRM being active. (We discuss how binding site separation and orientation alter *q_1_* later).

**Two pMad and one Tin binding site**

In this case, the three empty binding sites are assigned weight *[S_0_], [S_1_]* and *[S_2_],* where the first two are as above and *[S_2_]* is the empty site of the second pMad site. The possible states are now *[S_0_S_1_S_2_], [(AS_0_)S_1_S_2_], [S_0_(BS_1_)S_2_], [S_0_S_1_(AS_2_)],* *[(AS_0_)(BS_1_)S_2_], [(AS_0_)S_1_(AS_2_)], [S_0_(BS_1_)(AS_2_)]* and *[(AS_0_)(BS_1_)(AS_2_)]*. We consider the possibility that the state with three bound transcription factors has enhanced weight due to cooperative TF interactions between the bound pMad (denoted by β, Figure S7D). In effect, if *β>>*1 then the stability of the *[(AS_0_)(BS_1_)(AS_2_)]* state is significantly enhanced. The total weight of such a CRM is given by

*Z = [S_0_S_1_S_2_] + 2[(AS_0_)S_1_S_2_] + [S_0_(BS_1_)S_2_] + 2[(AS_0_)(BS_1_)S_2_] + [(AS_0_)S_1_(AS_2_)] + [(AS_0_)(BS_1_)(AS_2_)]*  (5)

Again using detailed balance, we find the probability of the CRM being active (*i.e.* pMad bound), given by

*p_on_ = (2a_0_+a_0_^2^+2αa_0_b_0_ + αβa_0_^2^b_0_) / (1+2a_0_+b_0_+a_0_^2^+2αa_0_b_0_ + αβa_0_^2^b_0_)* (6)

Applying the same assumptions as before (and considering only TF cooperative interactions as the relevant factors in CRM activity)

*p_on_ ≈ (2q_1_ + q_2_) / (1+2q_1_ + q_2_)*  (7)

where *q_1_* is as above and *q_2_* corresponds to the cooperative binding between adjacent pMad-Tin-pMad. If the second pMad binds independently of the pMad-Tin pair (*i.e. β=1*), then *p_on_≈2q_1_/(1+2q_1_)*. Although the model has a number of inputs (*e.g.* TF concentrations, binding rates), incorporating both pMad-Tin and pMad-Tin-pMad cooperative interactions into the model requires just two parameters (we discuss below how binding site separation and orientation alters these parameters). It is straightforward to extend this analysis to the Tin-pMad-Tin enhancer.

**Three pMad and three Tin binding sites**

For the CRMs with six TF binding motifs the number of all possible transcription factor binding configurations is large. However, for our purposes we need only consider configurations relevant for TF cooperative interactions. If the only cooperative interactions are between neighboring pMad-Tin pairs then

*p_on_ ≈ (5q_1_+ 6q_1_^2^+q_1_^3^) / (1+5q_1_+ 6q_1_^2^+q_1_^3^)* (8)

If pMad-Tin-pMad cooperative interactions play an important role in determining CRM activity then

*p_on_ ≈ (5q_2_+3q_1_^2^+2q_2_+3q_1_q_2_)/(1+5q_2_+3q_1_^2^+2q_2_+3q_1_q_2_)*  (9)

We also considered the possibility of more complex TF cooperative interactions, such as pMad-Tin-pMad-Tin-pMad. However, as detailed in the paper, interactions between three adjacent TFs (such as pMad-Tin-pMad) are sufficient to describe the experimental data well. Since the inclusion of further higher-order interactions comes at the price of additional parameters and it does not significantly improve the model fit we omit further discussion.

**Modeling CRM activity in the heart**

The above formulae are appropriate for heterotypic CRMs in the VM. For heterotypic CRMs in the heart the model needs to be adjusted. During this time, Tin itself is present in high concentration. Therefore, we include another possibility for a minimal TF cooperative interaction configuration, namely Tin-pMad-Tin. This change only alters the above equation for the three binding site CRM, where Eq. (7) becomes *2q_0_/(1+2q_0_)*. For the heart, a CRM is assumed to be active if at least one Tin site is bound.

**Accounting for binding site separation and binding site orientation**

The strength of the cooperative TF interactions depends on the overlap of the interaction domains between adjacent bound TFs. The actual topology of the protein binding domains is complex and so implementing the exact domain of protein interaction is not appropriate in the model. Instead, we approximate the protein binding domains as spheres and looked at the area of overlap between the spheres, Figure S7E. We assume that the area of overlap is proportional to the interaction strength. The sphere radius was taken to be *r*, with different effective radii for antisense and sense orientated transcription factors. For separation of the spheres by *d < 2r*, the phenomenological function describing the effective range of cooperative binding is given by

*w(d) = 1 - (d/2r)^2^* (10)

and is zero otherwise, Figures S7A and S7E. The distance dependence was introduced into the model via the *q_1_* and *q_2_* cooperative interaction terms: *q_1_(d) = q_1_(d=0)w(d)* and *q_2_(d) = q_2_(d=0)w(d)^2^*. As stated above, the difference between sense and antisense orientation of the Tin binding site was incorporated by altering the effective sphere size. In general, we found *r_sense_ < r_antisense_* - the role of binding site orientation is to alter the effective range over which bound proteins interact. Finally, we tested other forms for Eq. (10), such as *w(d) = 1 - (d/2r)^4^*. Our results are largely independent of the specific function, as long as *w(d)* is monotonically decreasing with *d* and is zero above some critical value of *d*.

**Calculating penetrance**

In the model we calculate the effective probability, *p_i_*, that an appropriate region of tissue displays activity for a particular CRM (labeled by *i*). For dorsally-aligned embryos there are four regions of relevant VM or heart, so the penetrance is then given by *P_i_ = 1 - (1-p_i_)^4^* (*i.e.* 1 - (Probability that no region shows CRM activity)). For laterally-aligned embryos, where only two regions are visible, this becomes *P_i_ = 1 - (1-p_i_)^2^*. The final measured penetrance for enhancer *i* was then

*P_i_ = (N_dorsal_(1-(1-p_i_)^4^) + N_lateral_(1-(1-p_i_)^2^))/N*  (11)

where *N_dorsal_, N_lateral_* and *N* are the number of dorsally-aligned embryos, ventrally-aligned embryos and total number of embryos respectively. In our data set we had approximately *N_dorsal_≈N_lateral_* and hence *P_i_ ≈ 1-(1/2)(1-p_i_)^2^(1+(1-p_i_)^2^).*

**Calculating expressivity**

The expressivity is the expected fraction of active regions in embryos that have at least some appropriate tissue-specific activity. For embryos aligned dorsally (*i.e.* four possible tissue-specific regions for both VM and heart, see Materials and methods) and assuming that the regions are independent of each other in the appropriate tissue, then for a particular enhancer with probability *p* of a region being active (all regions are assumed to have the same probability *p* of activation), the probability of only one active VM region observed, given that at least one is observed, is *g_0_ = (1 - p)^3^* (*i.e.* the probability that none of the other regions is active). The probability of exactly two regions being occupied is *g_1_ =3(1-p)^2^p* (since there are three ways of having one other region on and the other two off, given that the fourth region is already on). The probability of exactly three regions being occupied is *g_2_ =3(1 - p)p^2^* (since there are three ways of having two other regions on and the other off, given that the fourth region is on). The probability of all four regions on is *g_3_ =p^3^* (given that at least one is already on). For embryos aligned laterally, then *g_0_=(1-p)* and *g_1_ = p* as only two distinct regions are now visible.

The expressivity for enhancer *i*, *E_i_*, is the normalized expected number of regions: for dorsally-aligned embryos, *E_i_ = (1g_0_ + 2g_1_ + 3g_2_ + 4g_3_ ) / 4 = (1+3p)/4*; and for laterally-aligned embryos *E_i_ = (1g_0_+2g_1_)/2 = (1+p)/2*. Since the data set used to find the expressivity was small (typically only 16 embryos) we recorded the orientation of each embryo and used this when calculating expressivity to ensure the correct contribution from the above forms for *E_i_*.

**Model fitting**

Model fitting is done by minimizing the function

 (12)

where *P^(i^*^)^ and *E^(i)^* denote the penetrance and expressivity respectively from a CRM labeled by *i* and the sum is over all appropriate CRMs. The model was fitted only to data from the six TF motif pMad-Tin CRMs. The quality of fit was adjudged by the sum of the square of the residuals,

 (13)

where *P^(i)^_fit_* and *E^(i)^_fit_* are the theoretical values for the penetrance and expressivity respectively after parameter fitting.

Figure S8A, E show the model fits to the heterotypic CRM activity in the VM and heart respectively when only cooperative interactions between independent pMad-Tin pairs are considered. Figure S8F also shows the model fit to the heterotypic CRM activity in the heart when pMad-Tin-pMad cooperative interactions are taken as the fundamental unit. All model parameters are shown in Table S6.

**Supplemental References**

1. Halfon MS, Carmena A, Gisselbrecht S, Sackerson CM, Jimenez F, et al. (2000) Ras pathway specificity is determined by the integration of multiple signal-activated and tissue-restricted transcription factors. Cell 103: 63-74.

2. Xu C, Kauffmann RC, Zhang J, Kladny S, Carthew RW (2000) Overlapping activators and repressors delimit transcriptional response to receptor tyrosine kinase signals in the Drosophila eye. Cell 103: 87-97.

3. Bailey TL, Boden M, Buske FA, Frith M, Grant CE, et al. (2009) MEME SUITE: tools for motif discovery and searching. Nucleic Acids Res 37: W202-208.

4. Zhu LJ, Christensen RG, Kazemian M, Hull CJ, Enuameh MS, et al. (2011) FlyFactorSurvey: a database of Drosophila transcription factor binding specificities determined using the bacterial one-hybrid system. Nucleic Acids Res 39: D111-117.

5. Markstein M, Pitsouli C, Villalta C, Celniker SE, Perrimon N (2008) Exploiting position effects and the gypsy retrovirus insulator to engineer precisely expressed transgenes. Nat Genet 40: 476-483.

6. Sandmann T, Jensen LJ, Jakobsen JS, Karzynski MM, Eichenlaub MP, et al. (2006) A temporal map of transcription factor activity: mef2 directly regulates target genes at all stages of muscle development. Dev Cell 10: 797-807.

7. Zinzen RP, Girardot C, Gagneur J, Braun M, Furlong EE (2009) Combinatorial binding predicts spatio-temporal cis-regulatory activity. Nature 462: 65-70.

8. Karolchik D, Baertsch R, Diekhans M, Furey TS, Hinrichs A, et al. (2003) The UCSC Genome Browser Database. Nucleic Acids Res 31: 51-54.

9. Hertz GZ, Stormo GD (1999) Identifying DNA and protein patterns with statistically significant alignments of multiple sequences. Bioinformatics 15: 563-577.

10. Soding J (2005) Protein homology detection by HMM-HMM comparison. Bioinformatics 21: 951-960.

11. McGuffin LJ, Bryson K, Jones DT (2000) The PSIPRED protein structure prediction server. Bioinformatics 16: 404-405.

12. Sali A, Blundell TL (1993) Comparative protein modelling by satisfaction of spatial restraints. J Mol Biol 234: 779-815.

13. BabuRajendran N, Palasingam P, Narasimhan K, Sun W, Prabhakar S, et al. (2010) Structure of Smad1 MH1/DNA complex reveals distinctive rearrangements of BMP and TGF-beta effectors. Nucleic Acids Res 38: 3477-3488.

14. LaRonde-LeBlanc NA, Wolberger C (2003) Structure of HoxA9 and Pbx1 bound to DNA: Hox hexapeptide and DNA recognition anterior to posterior. Genes Dev 17: 2060-2072.

15. Chai J, Wu JW, Yan N, Massague J, Pavletich NP, et al. (2003) Features of a Smad3 MH1-DNA complex. Roles of water and zinc in DNA binding. J Biol Chem 278: 20327-20331.

16. Shi Y, Wang YF, Jayaraman L, Yang H, Massague J, et al. (1998) Crystal structure of a Smad MH1 domain bound to DNA: insights on DNA binding in TGF-beta signaling. Cell 94: 585-594.

17. Pettersen EF, Goddard TD, Huang CC, Couch GS, Greenblatt DM, et al. (2004) UCSF Chimera--a visualization system for exploratory research and analysis. J Comput Chem 25: 1605-1612.

18. Ay A, Arnosti DN (2011) Mathematical modeling of gene expression: a guide for the perplexed biologist. Crit Rev Biochem Mol Biol 46: 137-151.
